# Digital Platform to Provide Health Data Feedback for Neurorehabilitation Patients: User-Centered Development and Proof-of-Concept Usability Study

**DOI:** 10.2196/85072

**Published:** 2026-06-17

**Authors:** Nadine Domnik, Katarzyna Krasnopolska, Ramona Sylvester, Jens Bansi, Jaeyong Song, Roman Gonzenbach, Olivier Lambercy

**Affiliations:** 1 Department of Health Science and Technology, Rehabilitation Engineering Laboratory, ETH Zurich, Gloriastrasse 37/39, Zurich, 8092, Switzerland, 41 044 632 72 33; 2 Neurology Department, Kliniken Valens, Valens, Switzerland; 3 Department of Health, OST - Eastern Switzerland University of Applied Sciences, St. Gallen, Switzerland; 4 Future Health Technologies Programme, Singapore-ETH Centre, Singapore, Singapore

**Keywords:** neurorehabilitation, digital health, user-centered design, clinical decision-making, health data visualization, mobile health (mHealth), value based health care (VBHC), digital feedback application

## Abstract

**Background:**

An increasing amount of digital health data are being collected across rehabilitation settings, but their integration into routine clinical practice remains limited, despite its potential to motivate patients or inform clinical decision-making. Specifically, effective visualization and communication of assessment outcomes to both patients and health care practitioners (HCPs) represent a key gap in the neurorehabilitation practice.

**Objective:**

This study describes the development and evaluation of RehaLink (author ND, ETH Zürich), a proof-of-concept mobile app that delivers structured, interpretable feedback from conventional and technology-based assessments to neurorehabilitation patients and HCPs.

**Methods:**

The app was developed through a 3-step iterative co-design process involving 17 inpatients with multiple sclerosis and 15 HCPs from a single rehabilitation center. The app integrates a full battery of conventional assessments routinely conducted at the clinic, as well as digital health metrics from the Virtual Peg Insertion Test, a validated technology-based assessment of upper limb function, as a proof of concept for integrating technology-based assessment data into clinical workflows. Three structured feedback sessions were conducted, in which participants evaluated feedback types, visualization formats, and app usability using Likert-scale ratings, preference rankings, open-ended questions, and the System Usability Scale. Data were analyzed using descriptive statistics and directed content analysis.

**Results:**

Across all 3 sessions, progress bars and color-coded indicators were consistently preferred over text-heavy or abstract formats by both patients and HCPs. A persistent set of competing demands was observed, with participants requesting both visual simplicity and access to absolute values and normative comparisons. HCPs tended to underestimate patients’ preference for informative visualizations. The perceived value of structured feedback increased over the course of the study; patients’ median ratings rose from 4.0 to 5.0 and HCPs’ from 4.0 to 4.5 on a 5-point Likert scale. The resulting mobile app prototype demonstrated high usability, with patients achieving a mean System Usability Scale score of 93.6 (mean 6.4; best imaginable) and HCPs 80.9 (SD 8.1; good), according to established benchmarks.

**Conclusions:**

These findings demonstrate the feasibility and value of a co-designed digital feedback tool for neurorehabilitation. By combining conventional and technology-based assessment outcomes in an accessible, user-centered format, the app has the potential to enhance patient engagement, support clinical decision-making, and advance the implementation of value-based, personalized care.

## Introduction

During neurorehabilitation, both conventional and technology-based assessments are essential for diagnosing impairments, tracking progress, and tailoring treatments [1-5]. Standardized tools such as the Nine Hole Peg Test [6], the Timed Up and Go [7], and the Functional Independence Measure [8] are widely used [9]. Although they capture different domains of the International Classification of Functioning, Disability, and Health framework [10], they are subjective, time-consuming, and lack sensitivity [11-13].

Technology-based assessments address these limitations through automated data collection, improved objectivity, and greater sensitivity. Examples include (markerless) motion capture [14-16], wearable sensors [17], and robotic tools such as the Kinesiological Instrument for Normal and Altered Reaching Movement (KINARM) [18,19] or the Virtual Peg Insertion Test (VPIT) [20,21]. However, their outputs can be abstract, poorly validated, and difficult to integrate into clinical workflows [22].

Despite their potential to support the implementation of precision medicine, integrating both conventional and digital assessments systematically into clinical routine decision-making remains challenging. In many cases, assessment outcomes are difficult to synthesize and communicate effectively during patient consultations, which can limit their ability to enhance patient motivation, satisfaction, and adherence to treatment. This limited adoption is largely driven by the high effort required to collect, summarize, and interpret the data, which can outweigh the perceived benefit in many clinical contexts. Moreover, while the level of detail offered by technology-based assessments is valuable in research [23-25], its clinical relevance remains uncertain, and there is a lack of clear guidelines for selecting or applying metrics in practice [26].

Regular and well-communicated feedback on assessment outcomes has been shown to enhance patient motivation [27,28], support engagement in shared decision-making [29], and improve rehabilitation outcomes [30,31]. Likewise, transparent and interpretable data can support clinical decisions that are often otherwise driven by prior experience and/or subjective judgment [32]. To leverage the full potential of both conventional and technology-based assessments, their results must be accessible, interpretable, and actionable for both patients and health care practitioners (HCPs). Achieving this requires a systematic and collaborative approach to identify clinically meaningful outcome measures and establish health communication strategies that enhance their practical use in clinical routine [5,33,34].

Digital apps have the potential to bridge the gap between researchers, HCPs, and patients by enabling access to individualized health data and translating complex information into intuitive, user-friendly formats. By streamlining data collection and interpretation, these tools have the potential to support more frequent assessments and promote continuous communication of outcomes throughout the rehabilitation process. In recent years, the number of released health apps in areas such as mental health [35,36], consumer health [37], and fitness [38,39] increased rapidly. While many of these apps lack sufficient validation or fail to demonstrate clear benefits, others show strong potential to increase patient motivation and adherence [35,36,38,40]. A key factor in making digital health tools effective and engaging for both patients and HCPs is the active involvement of end users in the development process [41-46]. Applying user-centered design principles helps ensure that these tools are better aligned with the real needs of all stakeholders [3-5,40,41,46-50]. However, a key limitation is that many existing digital health apps are developed primarily for the consumer market rather than for integration into clinical workflows. As a result, their design often overlooks the specific requirements of clinical environments, limiting their adoption and utility in routine care. This gap is particularly evident in neurorehabilitation, where existing digital health record systems still lack effective features for summarizing and visualizing health-related data. Consequently, both HCPs and patients may lack access to user-friendly tools to monitor and understand rehabilitation progress.

This study describes a 3-step user-centered design process for developing a digital feedback app that displays digital health metrics from conventional and technology-based assessments to patients and HCPs along the neurorehabilitation journey. As the first step, we defined the need and requirements and evaluated expectations for such feedback together with HCPs. Through iterative rounds of discussions and structured interviews with both neurological patients and HCPs, we identified an optimal type and amount of information, along with the appropriate visualizations to display health data in an intuitive and engaging way. Our evaluation focused on 3 key aspects, including informativeness, understandability, and visual appeal. Building on this, we developed RehaLink (author ND, ETH Zürich), a proof-of-concept app that visualizes typical rehabilitation data collected during clinical routine assessments, including digital health metrics from the VPIT, and evaluated its usability. This work provides insights from end users—patients and HCPs—on how to meaningfully present assessment outcomes and include digital health metrics in neurorehabilitation settings that will open new avenues for empowering patients with their health data and support HCPs in decision-making towards precision medicine.

## Methods

### Conceptual Approach

**Figure 1.**
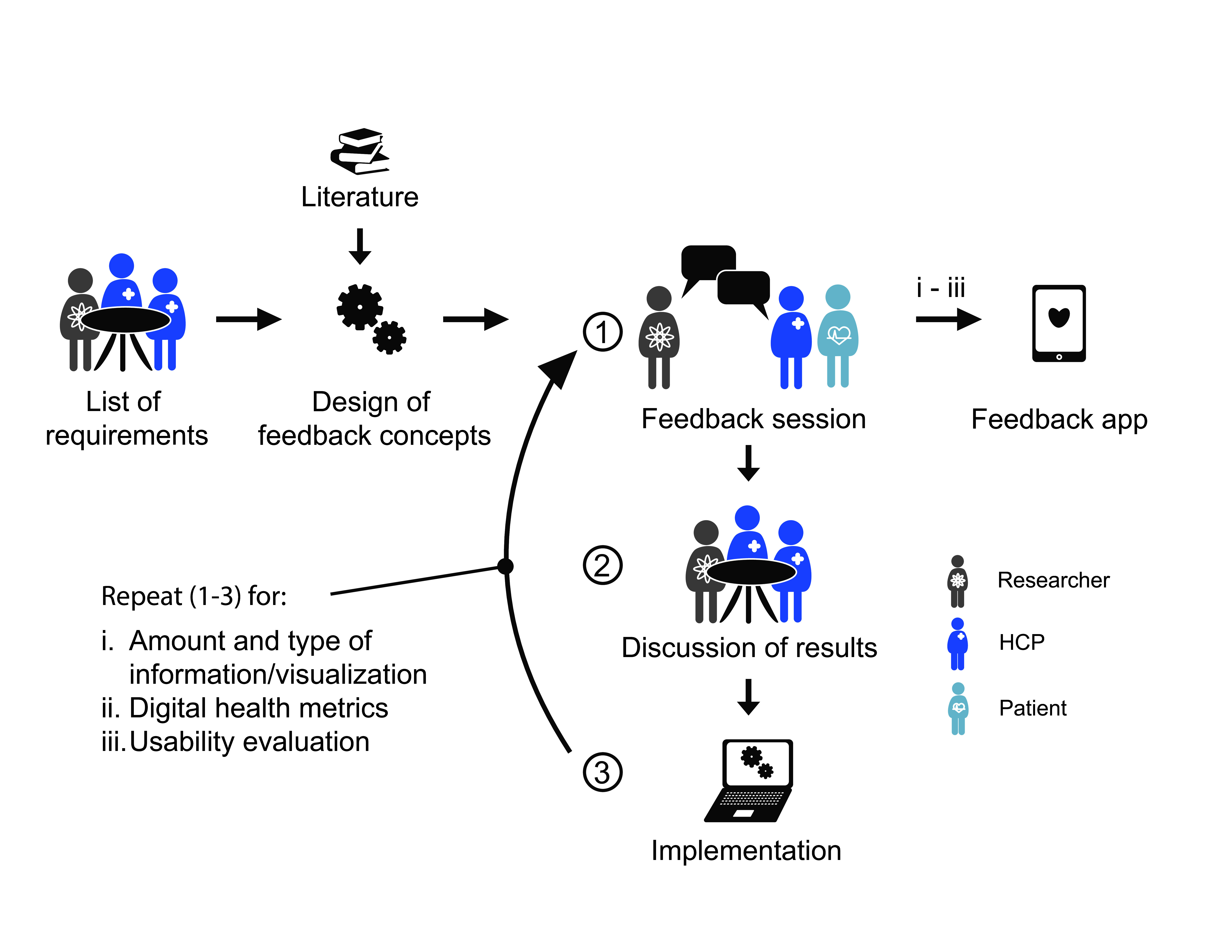
Overview of the co-design process for a digital feedback app for patients and health care practitioners (HCPs). The illustration shows a 3-step iterative approach guided by co-design principles. HCP: health care practitioner.

We used a structured co-design process to develop a digital app aimed at enhancing patient motivation and supporting clinical decision-making in neurorehabilitation. The initial requirements were identified through a multidisciplinary roundtable involving researchers from ETH Zurich and clinical staff from Kliniken Valens, including the heads of neurology, research, and therapy, as well as therapists with different backgrounds. The discussions focused on defining clinically relevant assessments, selecting the appropriate platform, and ensuring integration into routine care. The development process included three iterative feedback sessions with HCPs, designed to evaluate (1) the content and visualization of patient information, (2) the integration of digital health metrics, and (3) overall usability. Between sessions, targeted follow-up meetings with researchers and HCPs enabled systematic incorporation of feedback into subsequent development stages (Figure 1).

To ensure clinical relevance and align with clinical practice, the app was designed to include the full battery of conventional assessments routinely conducted at admission and discharge at the Kliniken Valens. To explore integration of digital health data, we included the VPIT, a well-validated tool for assessing upper limb function. The metrics derived from the VPIT are representative of the digital health data typically obtained from wearable sensors or robotic devices used in neurorehabilitation assessments. We implemented the app as a mobile solution for iOS, with an optional future compatibility across additional operating systems. At the time of this writing, the app is intended for use on tablets, as this offers greater practicality during patient consultations compared to stationary terminals.

### Participant Recruitment

Patients were recruited through purposive sampling from the inpatient neurology unit at Kliniken Valens. Although the app is intended for use across neurorehabilitation populations, patients living with multiple sclerosis (MS) were selected as the initial target group for this proof-of-concept study given the clinic’s large inpatient living with MS rehabilitation program that provides a readily accessible and well-characterized patient pool for this co-design process. Recruitment targeted eligible participants advised to inpatient rehabilitation with a diagnosis of MS and sufficient cognitive and communicative capacity to participate in structured feedback sessions. Eligible patients were identified by the treating clinical team through review of the inpatient list, selecting those with an MS diagnosis and sufficient cognitive and communicative capacity as judged by the attending clinicians. Participants provided informed consent under the institutional general consent framework. No formal inclusion or exclusion criteria were predefined, consistent with the exploratory, co-design nature of this work. Interested patients were approached by clinical staff and invited to participate voluntarily. HCPs were recruited through announcements during regular team meetings within the therapy and neurology departments of the Kliniken Valens.

The number of participants per session was determined pragmatically, as the primary aim of the feedback sessions was an iterative design refinement within a co-design process, rather than achieving qualitative data saturation in the traditional sense. Session recruitment concluded when the research team judged that sufficient stakeholder input had been obtained to inform the next design iteration, consistent with descriptions of co-design as an iterative, resource-bounded process [51-53]. Session sizes were consistent with recommendations for iterative, design-oriented evaluation, in which small samples have been shown to yield sufficient feedback for informing successive design iterations [54,55]. Nonetheless, qualitative responses in later sessions showed increasing thematic overlap with earlier feedback, suggesting that the key design-relevant perspectives had been captured within the sample.

### Conventional Assessments

The conventional assessments at Kliniken Valens were grouped into 6 categories, including endurance, mobility, strength, coordination, speed, and global health/function to support clearer interpretation in the app (Table S1 in Multimedia Appendix 1). These categories were based on the International Classification of Functioning, Disability, and Health framework and refined with HCP input.

### VPIT

The VPIT is a technology-aided assessment of upper limb function that integrates a haptic device with a virtual environment [20]. The task involves goal-directed reaching, in which users are instructed to insert nine virtual pegs into 9 holes displayed on a board as quickly and precisely as possible. During task execution, kinetic and kinematic data are continuously recorded. From this data, 10 digital health metrics are computed to quantify various aspects of movement quality, including smoothness, efficiency, accuracy, speed, and grip force control [21]. These metrics enable sensitive and objective characterization of upper limb impairments and have been validated in diverse patient populations, including individuals with MS [56,57], stroke [58], and ataxia [59,60]. Importantly, the VPIT served as a model for integrating digital health metrics into clinical feedback systems, offering a template for incorporating validated, objective assessments into user-facing tools. As such, it offers a clinically relevant and generalizable use case for exploring how complex sensor-based outputs can be effectively visualized and communicated to both patients and HCPs. Its integration into the co-designed digital feedback app supports our broader goal of making digital health data more accessible and actionable, fostering patient engagement and shared decision-making in neurorehabilitation. In collaboration with HCPs from Kliniken Valens, the VPIT metrics were grouped into the same 5 functional categories as the conventional assessments. An overview of the 10 core VPIT metrics, including their descriptions and associated categories, is provided in Table S2 in Multimedia Appendix 1.

### Structured Feedback Sessions

#### Overview

Three structured feedback sessions were conducted to iteratively evaluate key aspects of the app design, including information presentation, metric representation, and overall usability. Each session followed a standardized procedure lasting approximately 30-45 minutes, with 2 participants—either HCPs or patients—participating simultaneously. To minimize bias, all participants first received a neutral written introduction outlining the session’s purpose. In sessions 2 and 3, this was supplemented by a short video and an explanation of the VPIT.

Participants were then shown a series of feedback concepts and completed the structured questionnaires in MultimediaAppendices 2^7^. These included Likert-scale items (1=strongly disagree-5=strongly agree) [61], open-ended questions, and preference rankings of the concepts. Concepts were rated on motivational value, visual appeal, informativeness, and understandability. Additional items assessed the perceived usefulness of performance feedback—either for enhancing therapy motivation (patients) or supporting clinical decision-making (HCPs)—as well as attitudes toward technology-based metrics, using the VPIT as an example. Participants were encouraged to ask both technical and content-related questions throughout, ensuring a full understanding of the material.

After completing the questionnaire, participants could share additional thoughts in a brief open discussion. All reflections were manually transcribed, and quotes included in this manuscript were translated from German into English.

The feedback concepts were developed according to established user-centered design principles [41,45,48], balancing clarity and representativeness while minimizing bias during evaluation.

#### Feedback Session 1: Type and Amount of Information

The first structured feedback session focused on evaluating how different types and levels of information, as well as their visual presentation, influenced user perceptions of usefulness, clarity, and engagement. To reflect a realistic interaction flow, we structured the session around three conceptual screens: (1) a start screen summarizing overall therapy progress, (2) an assessment overview screen displaying all assessment outcomes, and (3) an assessment detail screen providing more in-depth information on a selected assessment outcome. The 3-level screen structure was designed to balance conciseness and depth, allowing users to quickly access key insights or explore detailed results as needed. Limiting the structure to 3 screens aimed to maintain a shallow navigation hierarchy, thereby enhancing usability in rehabilitation contexts, where cognitive and physical demands on users may be heightened [62].

For each screen, participants were shown a set of alternative feedback concepts that varied in content complexity and visual format. These included combinations of textual summaries, color-coded timelines, bar and line charts, spider plots, emojis, and progress bars. A text- and emoji-only version was also included in the first set to serve as a low-complexity baseline. These types of visualizations were selected for their common use in digital health or consumer apps, with bar and line graphs being the most prevalent in digital health contexts [63,64].

**Figure 2.**
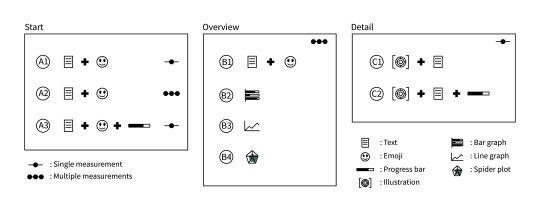
Illustration of feedback concepts shown during the first structured feedback session. The figure presents the 3 conceptual screens used to evaluate feedback design: the start screen, which provides overall performance summaries; the metric overview screen, which compares visualization types across multiple assessments; and the assessment detail screen, which presents in-depth explanations of single assessment outcomes. Each screen included multiple concepts (labeled 1-4) that varied in complexity, visual format, and content presentation.

An overview of the 3 screens and their respective feedback concepts is presented in Figure 2. This layered approach was designed to assess preferences across varying levels of information granularity and visual representation.

In addition to the concept-specific ratings, patients were asked whether feedback on assessments could enhance their motivation for therapy and which types of feedback they would prefer to receive. HCPs were asked whether feedback could support clinical decision-making and help motivate patients. Further, HCPs were asked if they think that the VPIT could be helpful for them to evaluate the upper limb function of the patients. The questions of whether patients think that structured feedback could enhance their motivation and whether HCPs think that it could support decision-making were asked before providing feedback concepts and again afterward, so that we get an unbiased opinion first. Both groups were also invited to indicate the kinds of technology-based assessment feedback they considered most meaningful.

#### Feedback Session 2: Presentation of Digital Health Metrics

The second structured feedback session focused on how digital health metrics could be effectively communicated to patients and HCPs through intuitive, engaging visual designs. To enhance understandability, the 10 VPIT metrics (Table S2 in Multimedia Appendix 1) along with overall task completion time were grouped into 5 lay-language metrics, including total time and speed, movement smoothness, accuracy, efficiency, and grabbing and holding. These categories were derived from the original research-based classifications and were adapted in collaboration with HCPs to ensure that terminology was intuitive and meaningful for both patients and HCPs. To support aggregation of metrics with different units and easier interpretation by nonexpert users, each VPIT metric was first normalized to a 0‐100 scale, where 0 represents the most impaired neurological subject in our database (excluding outliers), and 100 corresponds to the median performance of age-matched healthy controls. Without normalization, the raw values are too abstract and/or technical to convey meaningful feedback to patients or HCPs. The normalized metrics within each category were then averaged, without weighting, to create composite scores representing broader functional domains (eg, dimensionless log jerk and spectral arc length were combined under movement smoothness). This approach preserved metric validity while enhancing interpretability for end users. Details of the metric groupings are provided in the description column of Table S2 in Multimedia Appendix 1.

**Figure 3.**
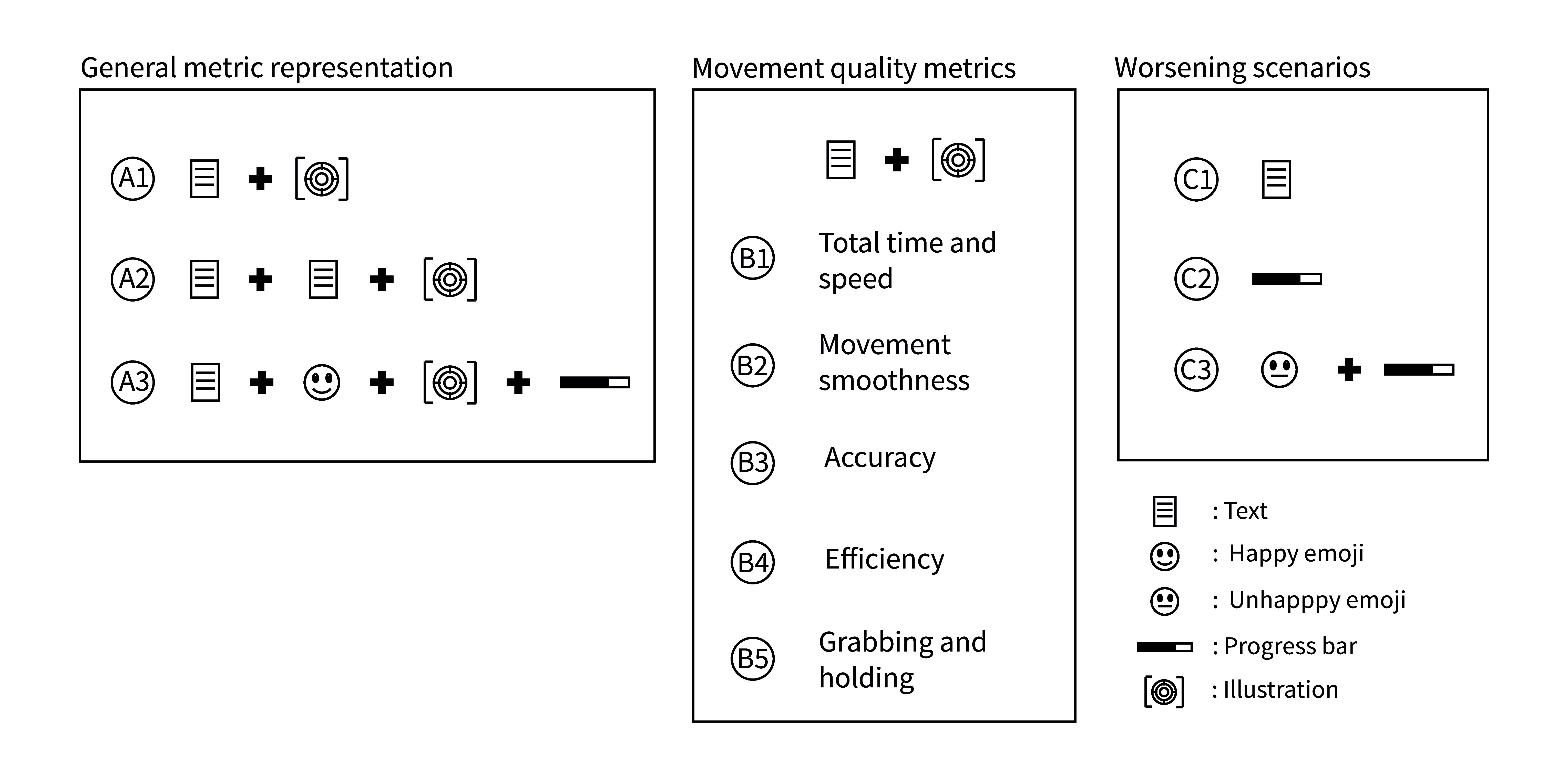
Overview of feedback concepts presented in the second structured feedback session. The figure summarizes 3 sets of visual feedback concepts shown to participants: Set 1 presents different designs for a single digital health metric (movement smoothness); Set 2 shows all 5 aggregated metrics with metric names, icons, visual illustrations, and explanations; and Set 3 explores how to communicate worsening outcomes, ranging from neutral to emotionally framed representations.

Participants were presented with three sets of feedback concepts (Figure 3), each designed to explore a different aspect of digital health metric feedback delivery.

Set 1 explored the general presentation of a single metric, using movement smoothness as an example. The 3 concepts varied in their level of explanation and visual complexity. Each included the metric name, an icon, and different combinations of visual elements such as color-coded bars (green for healthy and red for impaired), progress bars, emojis, and explanatory text.

Set 2 provided an overview of all 5 aggregated VPIT metrics. Each metric was shown with its name, icon, short descriptive text, and a corresponding illustration. This set aimed to assess whether the metrics were perceived as clear, useful, and engaging.

Set 3 addressed how to communicate worsening outcomes and included 3 visualizations with varying emotional tone and visual emphasis. The first concept displayed a neutral message (“No improvement”) without emojis or colored bars. The second used a green progress bar to indicate decline but excluded emojis and text. The third showed worsening results with an orange progress bar and an unhappy orange emoji. Each concept included an information button linking to an HCP-validated disclaimer explaining that performance changes may reflect normal day-to-day variation and should not be interpreted as clinically significant.

Participants rated each concept based on visual appeal, informativeness, and understandability. Additional open-ended questions captured qualitative impressions. Patients and HCPs were also asked whether they found the disclaimer clear and reassuring.

#### Feedback Session 3: Usability Evaluation of the App Prototype

In the third feedback session, participants evaluated the usability of a high-fidelity app prototype created using Figma (Figma, Inc) [65]. The prototype featured randomly generated data and allowed participants to freely explore its interactive components, including screen navigation and button functionalities. The use of randomly generated data for prototype usability testing is consistent with standard iterative user-centered design practice [41,42], in which interface usability is established prior to clinical validation. Further, instead of just illustrations for the VPIT metrics, we included animations.

Participants were instructed to interact with the app independently. HCPs were asked to imagine they were preparing for a consultation and tasked with reviewing a patient’s most recent assessment results, identifying any worsening trends, and checking the summary screen. Patients were instructed to imagine they had just completed a new assessment and were asked to explore their feedback, compare it with previous results, and interpret the meaning of their scores. Participants were encouraged to ask questions during the interaction if clarification was needed.

Following the exploration phase, all participants completed the System Usability Scale (SUS) [66] to evaluate overall usability. In addition, they responded to several targeted questions using 5-point Likert scales and open-ended questions. These assessed whether participants perceived structured feedback as valuable and if there is something to improve in the app design and implementation. Items also addressed the desire for comparative feedback (eg, performance relative to healthy individuals or other patients) and whether participants would like to celebrate personal milestones. Participants were also asked to suggest changes or propose features to add or remove.

### Data Analysis

#### Quantitative Analysis

Likert-scale responses were collected on paper and subsequently transferred to Python (version 3.13.2; Python Software Foundation) for analysis. No data exclusion or cleaning criteria were applied. For each item, the median and IQR were calculated separately for patients and HCPs. Median and IQR were chosen due to the ordinal nature of the data and their robustness to outliers, particularly in the context of small sample sizes. Accordingly, no inferential statistical testing was performed. Participants also ranked the feedback concepts within each concept set by assigning a unique rank to each concept, with no ties permitted. Median ranks were computed to identify overall user preferences for different content types and visual formats. Visualizations such as heatmaps and summary tables were created in Python to support interpretation.

#### Usability Assessment

SUS questionnaires were collected on paper and subsequently transferred to Python (version 3.13.2) for analysis. Raw SUS responses were converted into standardized scores (0‐100) following the standard scoring procedure [66]. Group-level means and standard deviations were calculated separately for patients and HCPs. Scores were interpreted using the adjective rating scale proposed by Bangor et al [67], ranging from “worst imaginable” (<50) to “best imaginable” (>90), to contextualize the usability findings.

#### Qualitative Analysis

Open-ended responses from all 3 feedback sessions were analyzed using direct content analysis. All responses were collected and analyzed in German. A single coder conducted multiple passes through the data, an initial pass to assign open codes to individual responses, followed by subsequent passes to group related codes into higher-level categories and identify recurring themes. An initial codebook was developed deductively based on the predefined questionnaire dimensions (eg, visual appeal, informativeness, understandability, and motivational value). Open-ended responses were then coded iteratively, with additional codes emerging inductively from the data and grouped into higher-level categories as recurring patterns were identified across participants. Coded themes were subsequently reviewed and discussed with all co-authors to ensure their plausibility and consistency with the broader dataset. Representative quotes were selected to illustrate key findings and translated from German into English by the first author (ND) for inclusion in the manuscript.

### Ethical Considerations

All participants were informed about the use of their data for scientific purposes and provided consent for further use of their data at entry to the Valens clinic. The responsibilities for this analysis were obtained by the Regional Ethics Committee of Eastern Switzerland (EKOS; EKOS 25/209). All procedures were conducted in accordance with the Declaration of Helsinki and the Swiss Human Research Act. All data collected were fully anonymized prior to analysis, and no personally identifiable information is reported in this manuscript. Participants received no financial compensation for their involvement.

## Results

### Participant Overview

A total of 17 neurological inpatients diagnosed with MS and 15 distinct HCPs from the Kliniken Valens participated in one of 3 structured feedback sessions. Several HCPs participated in more than one session, resulting in a total of 26 HCP participations. An overview of participant demographics and roles is provided in Table 1.

**Table 1.** Participant demographics across the 3 structured feedback sessions. The table summarizes the number of participants, their roles, and basic demographic characteristics for both patients and health care practitioners (HCPs) involved in each of the 3 structured feedback sessions.

Session and group		Participants, n	Age (years), mean (SD)	Gender (n)^,^	Role
Session 1					
Patients		5	40.8 (15.6)	Female: 2 Male: 3	—^a^
HCPs^b^		8	35.8 (10.4)	Female: 5 Male: 3	PT^c^ (n=3), ST^d^ (n=2), OT^e^ (n=2), HST^f^ (n=1)
Session 2					
Patients		6	56.5 (7.8)	Female: 1 Male: 5	—
HCPs		9	35.2 (9.3)	Female: 3 Male: 6	PT (n=5), ST (n=2), OT (n=2)
Session 3					
Patients		6	46.1 (14.8)	Female: 5 Male: 1	—
HCPs		8	33.1 (10.3)	Female: 1 Male: 7	PT (n=3), ST (n=2), OT (n=2), HST (n=1)

a Not available.

b HCP: health care practitioner.

c PT: physiotherapist.

d ST: sports therapist/scientist.

e OT: occupational therapist.

f HST: Health Science and Technology degree.

### Feedback Session 1: Type and Amount of Information

#### Overview

**Figure 4.**
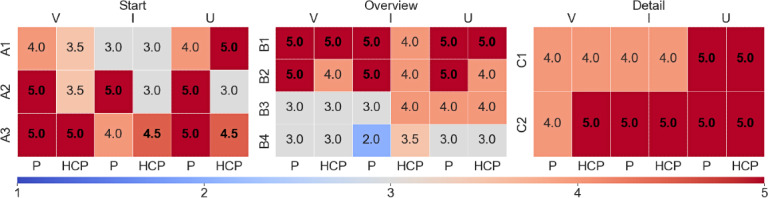
Median Likert-scale ratings for feedback concepts in feedback session 1. Participants rated 9 feedback concepts on 3 dimensions, visual appeal (V), informativeness (I), and understandability (U), using a 5-point Likert scale. Concepts are grouped by screen: start screen, overview screen, and metric detail screen. For the start screen and the detail screen, concepts are ordered by increasing informational complexity (A1-A3; C1-C2); for the overview screen, by increasing visual complexity (B1-B4). Ratings are shown separately for patients (P) and health care practitioners (HCP). Color intensity reflects the median score (red = favorable and blue = less favorable). Numbers in bold mark ratings above 4.5. I: informativeness; HCP: health care practitioner; P: patient; U: understandability; V: visual appeal.

The primary aim of this feedback session was to assess the preferred type and quantity of information to be presented to patients and HCPs within a digital feedback app. To reduce potential bias, participants were asked general questions about structured feedback both prior (pre) and then following their exposure to the different feedback concepts (post). The feedback concepts included various mockup screens designed to give participants an idea of how feedback might be presented; for example, through different data visualization formats such as bar charts, line graphs, spider plots, text, or even emojis. These concepts were created for a range of topics. In addition to presenting and evaluating feedback concepts, HCPs were asked whether feedback on completed assessments could aid clinical decision-making (pre: median 4.0, IQR 3.5‐4.0; post: median 4.0, IQR 4.0‐5.0), while patients were asked whether such feedback might increase their motivation to engage more consistently in therapy (pre: median 4.0, IQR 4.0‐5.0; post: median 4.0, IQR 4.0‐5.0). Median responses therefore remained consistent before and after viewing the feedback concepts. Additionally, HCPs were asked whether providing feedback to patients could enhance their motivation (median 4.5, IQR 4.0‐5.0) and whether digital health metrics such as VPIT-derived feedback would be useful in evaluating upper limb function (median 4.0, IQR 4.0‐5.0). Participants evaluated 9 feedback concepts across 3 screen types: the start screen, which summarized overall performance; the overview screen, which presented multiple assessment outcomes; and the detail screen, which displayed results for a single metric. All concepts were rated on visual appeal, informativeness, and understandability using Likert scales (Figure 4; Table S3 in Multimedia Appendix 1) and were ranked by preference.

#### Start Screen

The concepts evolved from a basic emoji-based design (concept A1: text+emoji) to a timeline-enhanced version (concept A2) and finally to a version featuring a progress bar (concept A3). Both Likert scale ratings and rankings showed that concept A3 (progress bar) received the highest overall scores. Patients ranked concepts A3 and A2 (timeline) the highest (median 2.0, IQR 1.0‐2.5 and median 2.0, IQR 2.0‐3.0, respectively), while HCPs rated concept A3 as their top choice (median 1.0, IQR 1.0‐1.5). Concept A3 also scored highest among both groups in terms of visual appeal, informativeness, and understandability. One patient remarked, “The progress bar makes it clear at a glance if I got better or worse,” and an HCP commented, “It is easily understandable for patients.”

#### Overview Screen

Concepts in this screen increased in visual complexity from text+emoji (B1) to bar plots (B2), line plots (B3), and spider plots (B4). HCPs ranked concept B3 slightly higher (median 2.0, IQR 1.0‐2.5), while patients gave similar rankings to concepts B2 (median 2.0, IQR 2.0‐2.0) and B3 (median 2.0, IQR 2.0‐4.0). Both groups ranked concept B4 the lowest. Several participants commented on the need for absolute values, stating, “We want to see how much improvement or worsening there is in absolute values or percentages.”

*If patients see norm values, they can compare what is good and where they need to improve. The same for us, we can then better compare where the patient is standing.*

[A health care practitioner (HCP)]

#### Detail Screen

Concept C2 (illustration+ text+progress bar) was ranked highest by both patients and HCPs (median 1.0, IQR 1.0‐1.0 and median 1.0, IQR 1.0‐2.0, respectively). Likert scores similarly favored this concept across all 3 dimensions. Participants made several comments on workflow, such as, “During clinical routine we don’t have time to study results, we want to directly be able to extract relevant information” and “Too many screens make the system not usable, it is too much effort.”

#### Open Feedback Themes

Participants provided additional comments regarding feedback preferences. Several HCPs mentioned the importance of wording; for example, “The language you use is important, it can be motivating for patients or not.” Concerns were raised about potential negative feedback effects; for example, “What about if their results get worse? Then it is not motivating anymore.” Patients commented on the importance of defining goals; for example, “What is my goal? I want it defined.” HCPs also suggested linking feedback to therapeutic recommendations, such as, “Add which therapy we can do to improve the specific results.” When asked what type of feedback they would like to receive about digital health metrics (eg, VPIT), HCPs emphasized graphical visualizations and clinically relevant parameters such as endurance, time, movement precision (eg, smoothness), and daily-life applicability. Several HCPs requested simple graphical trends, such as line charts, for tracking changes. Patients primarily focused on viewing their own progress over time, especially regarding speed and differences between admission and posttherapy sessions.

### Feedback Session 2: Presentation of Digital Health Metrics

#### Overview

**Figure 5.**
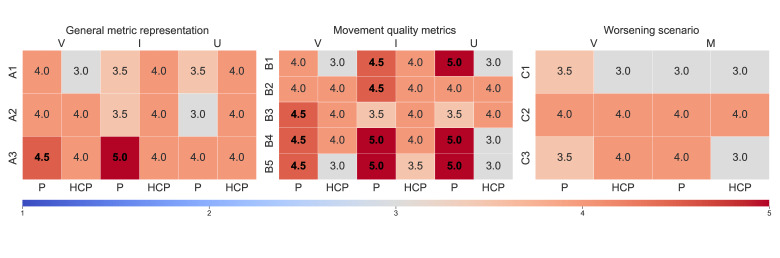
Median Likert-scale ratings for feedback on all concepts in feedback session 2. Participants rated 11 feedback concepts (A1-A3; B1-B5; C1-C3) from 3 sets: Set 1 (presentation of a single digital health metric), Set 2 (overview of all 5 grouped metrics), and Set 3 (how to present worsening results). Each concept was evaluated on visual appeal (V), each concept for Set 1 and 2 on informativeness (I), understandability (U), and on motivational (M) for Set 3 using a 5-point Likert scale. Ratings are shown separately for patients (P) and health care practitioners (HCP). Color intensity reflects the median score (red = favorable and blue = less favorable). Numbers in bold mark ratings above 4.5. I: informativeness; HCP: health care practitioner; M: motivational; P: patient; U: understandability; V: visual appeal.

The primary goal of this feedback session was to assess which digital health metrics patients and HCPs consider valuable and how these metrics could be visualized in an intuitive and engaging manner. In the second feedback session, participants evaluated 3 sets of feedback concepts, namely Set 1 focused on how to generally present digital health metrics using the example of movement smoothness; Set 2 focused on which metrics to present based on the condensed VPIT metrics; and Set 3 addressed how to communicate worsening of assessment outcomes. Each concept in Sets 1 and 2 was rated on visual appeal, informativeness, and understandability; each concept in Set 3 was rated on visual appeal and motivational aspects (Figure 5; Table S4 in Multimedia Appendix 1), while concepts in Sets 1 and 3 were additionally ranked by preference (Multimedia Appendix 1).

#### Set 1: General Metric Representation

Patients assigned the highest median rank to concept A2 (extended text+illustration; median 2.0, IQR 1.5‐2.0) and A3 (progress bar; median 2.0, IQR 1.0‐3.0), while HCPs most frequently ranked concept A3 (progress bar) as their top choice (median 1.0, IQR 1.0‐2.0). Among HCPs, concept A3 also received the highest Likert-scale ratings across visual appeal, informativeness, and understandability. Patients rated concepts A2 and A3 similarly on the understandability and informativeness dimensions. Qualitative feedback indicated varied preferences. One HCP described concept A2 as containing “too much information,” whereas concept A3 was described as “short, concise, and to the point.” Four additional HCPs commented that concept A3 was “simple, but understandable and very clear” and noted that it was “very well suited for patients.” Two HCPs suggested a combination of concepts A2 and A3. One patient commented that concept B2 included “too much text,” while concept A3 was “very well understandable.” Two other patients similarly stated that concept A3 was “very clear.”

#### Set 2: Digital Health Metrics

Participants evaluated 5 grouped digital health metrics, including time and speed (B1), smoothness (B2), accuracy (B3), efficiency (B4), and grabbing and holding (B5). Across all evaluated dimensions, patients generally provided higher Likert-scale ratings compared to HCPs. For visual appeal, accuracy (B3) and efficiency (B4) received the highest median ratings from both groups, with scores of 4.5 among patients and 4.0 among HCPs. In contrast, time and speed (B1) was rated lower, particularly by HCPs, with median scores of 4.0 for patients and 3.0 for HCPs. Regarding informativeness, efficiency (B4) and grabbing and holding (B5) were rated most favorably by patients, each achieving median scores of 5.0. HCPs rated all metrics slightly lower, with median scores around 4.0 and a lower rating for grabbing and holding (approximately 3.5). In terms of understandability, time and speed (B1), efficiency (B4), and grabbing and holding (B5) received the highest median scores among patients (5.0), while these metrics showed the lowest median scores among HCPs (3.0). Participant comments were specific to individual visual elements, with several suggestions for refinement. For example, one HCP proposed adding a tortoise and rabbit to the speed visualization to increase clarity.

#### Set 3: Worsening Scenarios

Participants evaluated 3 concepts for communicating worsening outcomes: C1 (text only), C2 (progress bar), and C3 (emoji+progress bar). Among patients, C2 received the highest median ranking (median 1.5, IQR 1.0‐2.0), while HCPs preferred C3 (median 2.0, IQR 1.0‐2.0). C1 was ranked lowest by both groups (median 3.0, IQR 2.0‐3.0). Likert-scale ratings showed that concepts with visual elements (C2 and C3) were generally rated higher in visual appeal, informativeness, and understandability compared to the text-only C1. Across most dimensions, patients gave higher ratings than HCPs. Participant comments added context; HCPs described C3 as a “good visualization,” appreciating the emphasis on positive aspects, though some felt the worsening was shown too strongly. Several suggested revising the emoji design, eg, using “neutral-colored, smiling emojis.” For C1, HCPs stressed the need for quantitative data, “always show values,” and “we always want to see exact values.” Patients also preferred visuals. For C3, they liked the color-coded improvements but recommended neutral smiles. One noted, “too much indicated the negative parts.” Regarding C1, a patient said, “I don’t want text, it is too negative. I prefer emojis and colors.” Participants also rated a text explanation stating that worsening results may reflect normal daily fluctuations. Understandability was rated at a median of 4.0 (IQR 3.0‐4.5) by HCPs and a median of 4.5 (IQR 3.5‐5.0) by patients; the median motivational value was 4.0 (IQR 3.0‐4.0) and 4.5 (IQR 4.0‐5.0), respectively. HCPs suggested making the message more encouraging and replacing “measurement variability” with relatable terms like environmental or daily performance factors. Patients asked for simpler language and generally found the text helpful. General feedback about the app development was also positive. One HCP noted, “The interface is a very nice opportunity for everyone; I am glad to be part of the optimizing process,” while a patient commented, “exciting project with a lot of informative insights into the therapy progress.”

### Feedback Session 3: Usability Evaluation of the App Prototype

**Figure 6.**
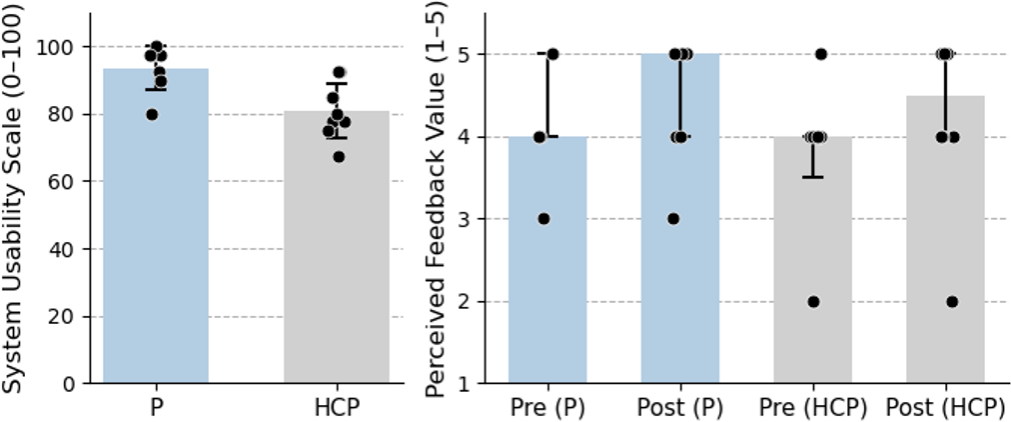
Evaluation of app usability and perceived value of structured feedback. The left panel shows System Usability Scale (SUS) scores for the app prototype as rated by patients (P) and health care practitioners (HCPs) in feedback session 3. The right panel presents pre–post ratings on the perceived value of structured assessment feedback in clinical routine, separately for patients and HCPs. Specifically, patients were asked if structured feedback about performed assessments could enhance their motivation to perform therapy on a more regular basis, and HCPs were asked if such feedback could aid them in decision-making processes. Patients and HCPs rated feedback value on a 5-point Likert scale before exposure to the feedback concepts (feedback session 1) and again after app testing (feedback session 3). Bars for the SUS score represent group means; error bars indicate SDs. Bars for the perceived value of structured feedback represent medians, error bars represent IQRs. Individual responses are shown as jittered black dots. HCP: health care practitioner; P: patient.

The main goal of this feedback session was to evaluate the usability of the first functional app prototype. During this final structured feedback session, participants interacted with the fully functional app prototype, which featured short animations in place of static illustrations to visualize digital health metrics. Usability was evaluated using the SUS. Patients reported a mean SUS score of 93.6 (SD 6.4), corresponding to “best imaginable” usability, while HCPs reported a mean score of 80.9 (SD 8.1), corresponding to “good” usability, according to established benchmarks [67] (Figure 6). Participants were also invited to suggest improvements to the prototype. One HCP requested the inclusion of absolute values alongside percentages in performance bars: “I would like to see an absolute number with units in every bar, not only the percentage indicating improvement or decline.” Another HCP highlighted the clarity of the interface, stating:

*The implementation of the user interface is really nice. It shows just the right amount of key information, it’s super easy to get a good overview.*

[An HCP]

Further suggestions included:

*Consider adding brief explanations when milestones are not reached, or positive reinforcement when progress is made. I’d also like to see training recommendations as videos, such as: ‘Watch this video again to improve parameter A/B/C.’*

[An HCP]

Another comment was:

*Could the system automatically assign milestones based on initial assessments? And for chronic patients, could past data from earlier years be integrated?*

[An HCP]

Patients also contributed feedback. One commented, “Compliment! There’s nothing I would want to change.” Another noted, “Can you include both the absolute values and percentage improvements in the bars?” Participants were asked to rate their perceived belief in the potential value of structured feedback in a clinical setting, reflecting attitudinal measures of perceived utility rather than demonstrated clinical benefit. Patients were specifically questioned on whether they believed such feedback could enhance their motivation during therapy, while HCPs were asked if it could aid clinical decision-making. This question was previously posed to participants of feedback session 1, before any feedback concepts were introduced, enabling a pre-post comparison. Median ratings from patients increased from 4.0 (IQR 4.0‐5.0) to 5.0 (IQR 4.0‐5.0), while HCPs’ median ratings rose from 4.0 (IQR 3.5‐4.0) to 4.5 (IQR 4.0‐5.0). Additionally, HCPs rated the potential of structured feedback to enhance patient motivation at a median of 4.5 (IQR 4.0‐5.0). Participants were also asked whether they would find value in performance comparisons to (1) age-matched healthy individuals or (2) other patients with similar conditions. Patients rated comparisons to healthy individuals at a median of 5.0 (IQR 5.0‐5.0) and to other patients at a median of 4.0 (IQR 3.0‐5.0). HCPs assigned corresponding median ratings of 4.0 (IQR 4.0‐4.5) and 3.0 (IQR 2.0‐4.0). Finally, the perceived benefit of celebrating therapeutic milestones within clinical settings was evaluated. Patients rated this concept at a median of 4.0 (IQR 3.0‐5.0), while HCPs provided a slightly lower median rating of 3.5 (IQR 3.0‐4.5).

### Cross-Session Themes

Across all 3 feedback sessions, several cross-cutting themes emerged. First, participants consistently preferred visually guided formats, particularly progress bars and color-coded indicators, over text-heavy or abstract representations such as spider plots or emoji-only designs, a pattern that was evident across all sessions and both stakeholder groups. Second, a set of competing demands was observed throughout: both patients and HCPs repeatedly requested access to absolute values and normative comparisons alongside simplified visuals. Third, the appropriate communication of worsening outcomes was a persistent concern across all sessions, raised by both groups from the earliest conceptual discussions through to the final prototype evaluation, ultimately informing the adoption of a neutral, color-coded feedback strategy without emotionally loaded symbols. Fourth, the perceived value of structured feedback grew across the course of the study, with both patients and HCPs rating its potential benefit more highly after exposure to the app prototype than before. Fifth, a consistent divergence in assumptions between patients and HCPs was observed. HCPs tended to recommend simpler formats out of concern that more detailed visualizations might overwhelm patients, while patients themselves consistently expressed a preference for richer, more informative representations.

### Final Implementation of the Digital Feedback App

The final RehaLink app incorporated input from both patients and HCPs across all 3 structured feedback sessions. It was implemented as a mobile iOS app with 2 distinct user interfaces, one for patients and one for HCPs. The primary difference lies in data management functionality, which is available only to HCPs. This includes the ability to create new patient accounts and manage user credentials, such as resetting passwords.

The app was developed using React Native (version 15.1.3; Meta Platforms) [68], leveraging the Expo framework (version 52.0.23; Expo) [69] to facilitate efficient cross-platform development, deployment, and maintenance. It was optimized for tablet use to ensure seamless integration into clinical workflows, including bedside consultations. The backend infrastructure was implemented with Django (version 5.1.5; Django Software Foundation) [70] and a structured SQLite3 (version 3.45.3; Hwaci) database [71], providing robust mechanisms for secure data storage, user authentication, and real-time data synchronization.

**Figure 7.**
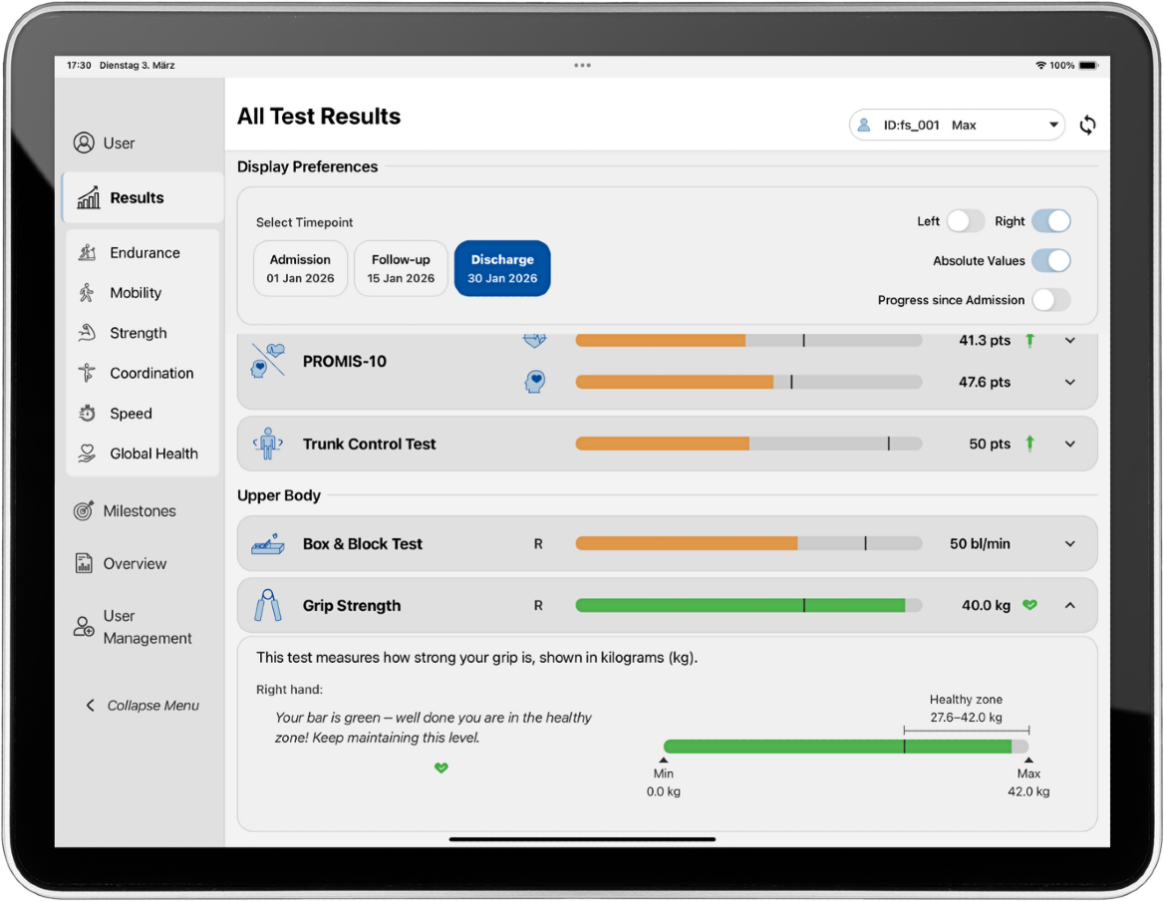
Final feedback app. Screenshot of the final RehaLink interface. The app is in normative mode; that is, shown progress bars indicate the users’ functionality compared to an assessment-specific normative population, including age, gender, and handedness factors. Drop-down menus provide assessment and visualization explanations. Assessments are grouped by body region and can be filtered by functional domain (left panel). The left panel can be collapsed to show icons only.

Data from technology-based assessments (eg, the VPIT) is automatically analyzed and then visualized in the app interface. By contrast, data from conventional assessments is currently extracted manually from the hospital information system and exported to the app. The final digital feedback app integrates all iterative refinements from the 3 performed structured feedback sessions (Figure 7).

Following session 1, findings were reviewed in a joint discussion with HCPs, resulting in a shared visual interface for both patients and HCPs to support use during consultations, with the HCP interface additionally including a patient selector and a user management tab. Navigation depth was reduced from 3 to 2 levels, with a login screen leading directly to an assessment overview. Assessments were grouped into upper body, lower body, and general categories, with a sidebar panel enabling filtering by functional domain (eg, strength and coordination) and access to personal health data (eg, BMI and weight) and the 3 primary therapy goals defined at admission. Color-coded bar charts were selected as the primary visualization format, supplemented by emoji-based indicators where appropriate.

Following session 2, a standardized format was introduced for digital health metrics, combining a recognizable icon, concise descriptive text, and an on-demand animated visualization illustrating key performance features (eg, jerky vs smooth movements). The VPIT metrics were visually aligned with conventional assessments, and all entries were supplemented with icons to support faster navigation. A left-right toggle was added for bimanual assessments to reduce visual clutter. Performance declines are indicated solely through color changes, without emotive symbols, accompanied by an explanatory note clarifying that fluctuations may reflect normal day-to-day variability. An overview panel was added displaying all assessment results in tabular format, including the patient’s absolute values, the corresponding normative reference values, and the minimal detectable change (MDC) or minimal clinically important difference (MCID) thresholds as explicit columns. Values falling outside the normative range are highlighted in bold red; directional arrows indicate whether changes exceed the MDC or MCID threshold, flagging clinically significant improvements or deteriorations. This panel supports rapid clinical review and is designed to serve as a printable summary.

Following session 3, information buttons were replaced with expandable drop-down menus providing on-demand contextual explanations. VPIT metrics were grouped into a single assessment block with distinct subcategories to prevent visual overemphasis. Value labels were added next to performance bars, available in both absolute and percentage formats with a user toggle between the two. A tab-based navigation system was introduced to enable longitudinal browsing across assessment time points, with the most recent results displayed by default.

Two display modes were implemented. The progress mode, shown by default, displays changes in performance relative to the patient’s baseline at admission, using a color-coding scheme based on the MDC or, where unavailable, the MCID. Improvements beyond the threshold appear as green bars extending right, changes within the threshold range in orange, and deteriorations beyond the threshold in red extending left. The normative mode maps each outcome to a 0‐100 scale referenced against an age-, gender-, condition-, and handedness-matched healthy population, where 100 represents the median healthy performance and the lower bound of the healthy range is defined as the median minus 2 SDs. Scores within the healthy range appear in green, values below the healthy threshold but above 50% in orange, and scores below 50% in red. Normative values for the VPIT were derived from a healthy reference database (n=120); norm values for conventional assessments were taken from previously published reference data [6,9,72-79]

To promote accessibility and personalization, the app includes user-adjustable settings such as dark mode, scalable font sizes, and high-contrast color schemes. Users can additionally hide assessment entries for tests that have not yet been performed, reducing visual clutter and cognitive load. Further, the default displayed unit (absolute vs percentage), hand side, and mode (normative vs progress) can be set for each user account. The interface is available in 4 languages, German, French, Italian, and English, to reflect the multilingual reality of Swiss rehabilitation settings.

## Discussion

This study presents a 3-step, iterative, user-centered approach to designing a digital app that delivers structured feedback on assessment results to patients and HCPs in neurorehabilitation. After defining clinical and user requirements, we conducted three structured feedback sessions to evaluate: (1) the type, amount, and visual format of information presented; (2) the integration and representation of digital health metrics from technology-based assessments; and (3) the usability of a digital app prototype delivering this feedback. The final application presents assessment outcomes in a co-designed format tailored to both stakeholder groups. Likert-scale ratings indicate a growing perceived value across all sessions.

The motivational and clinical benefits of personalized feedback are well supported in rehabilitation literature. Moreover, feedback that is timed, personalized, and performance-related improves engagement and motor outcomes and reduces patient anxiety [27,28,30,31]. While prior work mainly focused on real-time feedback during therapy, our app delivers post-assessment feedback. Nevertheless, similar benefits are expected, including increased motivation, better understanding of progress, and enhanced shared decision-making. Structured feedback has also been recognized as a tool for more evidence-based, value-based health care (VBHC) [32], which our co-designed digital app supports.

To ensure these expected benefits translate effectively into practice, our main focus was to evaluate how feedback is presented to users. Users preferred clear visuals such as bar charts with optional, layered explanations over more complex formats like line or spider graphs, which can be harder to interpret [63,64]. Beyond confirming general preferences for visual simplicity, our findings provide context-specific insights into why particular visualization formats failed in this neurorehabilitation setting. Spider plots were consistently ranked lowest by both groups, likely because radial layouts mask the directionality and magnitude of change, information that is particularly relevant for patients tracking rehabilitation progress over time. Text-only formats were rejected not only because they lacked visual appeal, but also because participants associated plain text with negativity, particularly when communicating worsening outcomes, consistent with prior findings on the psychological impact of health data framing [80,81]. Emoji-only formats failed because both groups considered quantitative information essential for meaningful interpretation, a finding that contrasts with their common use in consumer health apps. Extended text formats were specifically rejected by HCPs due to information overload in time-constrained clinical workflows, while patients were more tolerant of detailed explanations, reinforcing the divergence in informational needs between the 2 groups. Importantly, this divergence itself represents a finding that extends beyond established heuristics; HCPs consistently underestimated patients’ wishes for detailed, quantitative visualizations, suggesting that clinician judgments about patient interface preferences may be systematically biased toward oversimplification. More broadly, while existing literature addresses specific aspects of health data visualization in isolation, such as general patient preferences for chart types or usability of consumer health apps, our study takes a more comprehensive approach. Specifically, it is distinctive in evaluating the full pipeline from information selection and visualization design through to prototype usability, simultaneously engaging both patients and HCPs in a rehabilitation-specific context centered on the presentation of assessment outcomes. To our knowledge, no prior work has examined this complete process in this clinical setting, particularly with the integration of technology-based assessment metrics alongside conventional clinical assessments.

In contrast to many digital health tools that offer static or device-specific reports, our digital feedback app applies strategies like expandable content design and visual simplification [64] to preserve clinical utility without sacrificing usability. Compared to typical device-generated reports, our solution emphasizes personalization, clarity, and emotional neutrality. These preferences align with usability literature, especially for patients and older adults, key populations given the increasing prevalence of age-related neurological disorders. They tend to favor icon-driven navigation, high-contrast layouts, and intuitive visuals [46,49,50,82]. In line with these recommendations, RehaLink incorporates adjustable font sizes, high-contrast color options, and dark mode support, features shown to improve readability and reduce visual fatigue in older and neurologically impaired users [49,50]. The observed divergence between HCP assumptions and patient preferences underscores the importance of iterative, inclusive stakeholder engagement throughout the design process. Features like toggleable views—between absolute and normalized scores or between progress and normative benchmarks—allowed personalization without cognitive overload. These solutions addressed users’ varied informational needs while preserving interpretability and clinical relevance. Ramesh et al [83] used a symbolic, metaphor-driven approach that omitted numerical data and involved patients only at the final evaluation stage. In contrast, our participants were engaged throughout the full design process and explicitly requested access to raw scores. By combining quantitative metrics with intuitive visuals, our tool meets diverse informational needs and promotes transparency. Moreover, our larger, more diverse sample supports a broader generalizability. A key design insight was the need to balance simplicity with sufficient depth.

These design preferences not only influenced visual choices but also highlighted the importance of emotional tone and clinical context in delivering feedback. Participants were not just passive recipients of information but active stakeholders who were concerned with how their performance data were presented, especially when it involved sensitive or potentially discouraging results. As such, our co-design process extended beyond aesthetics to encompass the psychological and clinical dimensions of feedback delivery. While emojis were appreciated for reinforcing positive progress, participants favored more neutral, color-coded indicators and supportive language when feedback indicated worsening. This reflects prior findings that uncontextualized or overly negative feedback can increase patient anxiety [80,81] and should be carefully framed. Co-design allowed visual elements to be emotionally contextualized—emojis for improvement, neutral tones for decline—supporting engagement and comprehension. To ensure appropriate support, this digital feedback app is designed for use during patient consultations rather than in isolation.

In addition to emotional sensitivity, participants expressed a desire for actionable guidance and goal-setting tools. Patients valued reassurance during setbacks and the ability to track their own progress, while HCPs stressed the importance of clinically meaningful metrics and normative comparisons. This aligns with evidence supporting goal setting as a key motivator [27,84] and broader recommendations for embedding digital tools within VBHC frameworks [26]. Despite VBHC’s potential, its implementation remains challenging due to fragmented infrastructure and limited outcome tracking [3,33].

By combining conventional and digital health metrics in a co-designed tool, our approach addresses these gaps and has the potential to support data-driven, patient-centered care. A central aim of this work was to demonstrate how digital health metrics can be meaningfully integrated into clinical routines. Digital health metrics, such as those exemplified by the VPIT, contribute objective, fine-grained, and repeatable measurements that can enhance the sensitivity and clinical utility of outcome tracking [11,21,56,59]. Their standardized format allows for automated longitudinal monitoring, eliminating the need for manual digitization or additional data processing, and supports meaningful comparisons across patients and time points. While the VPIT specifically targets upper limb sensorimotor function, its metric design is conceptually aligned with other technology-based assessments, such as those using wearable sensors for upper limb monitoring [85,86]. These approaches share the goal of capturing movement quality and functional performance through quantifiable, reproducible measures. Moreover, the principles guiding the presentation and interpretation of VPIT metrics are broadly applicable to other rehabilitation domains, for example, including lower limb function and cognitive assessments, where a wealth of digital health metrics has been proposed [87-90]. As such, the use of digital health metrics in feedback tools offers significant potential to advance VBHC by improving outcome transparency, personalization, and clinical efficiency.

The final digital app, refined through iterative feedback, achieved high usability scores from both stakeholder groups (SUS: patients 93.6, SD 6.4; HCPs 80.9, SD 8.1). Users highlighted ease of navigation, clear interface design, and appropriate feedback granularity. Importantly, even participants with low digital literacy reported no major interaction issues, supporting the tool’s accessibility. Implementation feedback prioritized interoperability with electronic health records, inclusion of therapy recommendations, and milestone tracking. These additions, including automatic goal setting and benchmarking, enhance clinical integration and self-management.

While promising, this study is subject to several limitations. The patient cohort consisted only of persons with MS, recruited from a single neurorehabilitation center, which represents a limitation with respect to the direct generalizability of findings to other neurorehabilitation populations such as stroke or traumatic brain injury. However, several aspects of the study support broader applicability. First, no design decisions were specific to MS or restricted by condition-specific cognitive or motor profiles; the only practical prerequisite for app use is sufficient cognitive capacity to interact with a tablet-based interface, which applies across neurorehabilitation populations. Second, the HCPs involved were diverse neurorehabilitation therapists, and the conventional assessments integrated into the app are routinely used across neurorehabilitation conditions. Third, the VPIT, used here as a proof of concept for technology-based assessment integration, has been validated in individuals with different neurological disorders, such as MS, stroke, and ataxia. Moreover, while this evaluation was conducted using mock data, future studies should assess the app’s real-world impact on clinical decision-making, patient adherence, and therapeutic outcomes. The clinical utility of the selected conventional and technology-based assessments also remains to be established through routine use and empirical testing. Additionally, interpreting the digital health metrics, particularly their prognostic relevance and implications for therapy adaptation, requires larger datasets and further investigation. Understanding how these metrics translate into actionable clinical decisions is crucial for the tool’s long-term adoption and effectiveness. A limitation of the implementation is that data from conventional assessments requires manual extraction from the hospital information system to be exported to the app. While this introduces an additional workflow step, direct integration with clinical management systems was out of the scope of this work and not feasible at this stage of development due to data protection and information technology security constraints, which commonly apply in clinical settings. Should the ongoing evaluation demonstrate sufficient clinical benefit, integration of the app into the clinic’s existing information technology infrastructure is planned, which would enable automated data transfer and eliminate this manual step.

In summary, we demonstrate the feasibility and value of a co-designed tool for rehabilitation. Through structured feedback and iterative design, we developed a digital app that aligns with clinical workflows, enhances patient engagement, and has the potential to advance digital health integration. While these findings are promising, especially in terms of usability and stakeholder acceptance, further research is needed to evaluate the tool’s impact in real-world settings, across broader patient populations, and in fully integrated clinical infrastructures. Our results offer a foundational step toward embedding patient-centered feedback systems into clinical routine, with potential to support data-driven decision-making and VBHC.

## Supplementary material

10.2196/85072 Multimedia Appendix 1 Overview of all performed clinical assessments, VPIT metrics and median and IQR for participant ratings.

10.2196/85072 Multimedia Appendix 2 Patient feedback questionnaire 1.

10.2196/85072 Multimedia Appendix 3 Therapist feedback questionnaire 1.

10.2196/85072 Multimedia Appendix 4 Patient feedback questionnaire 2.

10.2196/85072 Multimedia Appendix 5 Therapist feedback questionnaire 2.

10.2196/85072 Multimedia Appendix 6 Patient feedback questionnaire 3.

10.2196/85072 Multimedia Appendix 7 Therapist feedback questionnaire 3.

## Abbreviations

EKOS: Ethics Committee of Eastern Switzerland
HCP: health care practitioner
KINARM: Kinesiological Instrument for Normal and Altered Reaching Movement
MCID: minimal clinically important difference
MDC: minimal detectable change
MS: multiple sclerosis
SUS: System Usability Scale
VBHC: value-based health care
VPIT: Virtual Peg Insertion Test
