# Supplementary material for: Digital Platform to Provide Health Data Feedback for Neurorehabilitation Patients: User-Centered Development and Proof-of-Concept Usability Study
Source: JMIR Rehabil Assist Technol. 2026 Jun 17;13:e85072. doi: 10.2196/85072 (PMC13274913; doi:10.2196/85072)
Supplement: Multimedia Appendix 1 [file rehab-v13-e85072-s001.pdf]

Supplementary Data

Supplementary Table S1. Overview of routine clinical assessments at Kliniken Valens. This table summarizes standardized assessments conducted upon admission and discharge of neurological patients at Kliniken Valens. Each assessment is briefly described and categorized according to its clinical purpose. Abbreviations: ADL, activities of daily living; QoF, quality of life; MS, Multiple Sclerosis

Assessment	Description	Category
Cumulative Illness Rating Scale (CIRS) [10]	Evaluation of impairment severity across multiple organ systems	Global health/function
Functional Independence Measure (FIM) [7]	Evaluation of functional independence in performing ADL	Global health/function
EQ-5D [3]	Measurement of health-related QoF	Global health/function
Patient-Reported Outcome Measure Information System (PROMIS-10) Global Health [5]	Assessment of global physical, mental, and social health through 10 items	Global health/function
Timed Up and Go (TUG) [13]	Evaluation of basic mobility, balance, and fall risk	Mobility
Functional Ambulation Categories (FAC) [6]	Assessment of walking ability	Mobility
Trunk Control Test (TCT) [2]	Evaluation of basic trunk movements	Mobility
10-Meter Walk Test (10MWT) [14]	Walking speed over 10 meters	Mobility
2-Minute Walk Test (2MWT) [1, 8]	Walking distance over two minutes	Mobility
Expanded Disability Status Scale (EDSS) [9]	Assessment of disability in MS	Mobility
6-Minute Walk Test (6MWT) [4]	Walking distance over six minutes	Endurance
Box & Block Test (BBT) [12]	Assessment of gross dexterity	Coordination
Jamar Hand Dynamometer [11]	Measurement of grip strength	Strength

Supplementary Table S2. Overview of the ten digital health metrics of the Virtual Peg Insertion Test (VPIT). This table summarizes the core metrics computed from kinetic and kinematic data recorded during the VPIT. The task involves multiple movement phases - transport, return, and peg or hole approach - during which these metrics are extracted. Each metric is categorized according to the functional grouping co-developed with healthcare practitioners.

Assessment	Description	Category
Dimensionless Log Jerk Transport	Movement smoothness	Coordination
Dimensionless Log Jerk Return	Movement smoothness	Coordination
Spectral Arc Length Return (SPARC)	Movement smoothness	Coordination
Path Length Ratio Transport	Movement efficiency	Coordination
Path Length Ratio Return	Movement efficiency	Coordination
Jerk Peg Approach	Endpoint Error	Coordination
Max. Velocity Return	Movement Speed	Speed
Number of Force Peaks Transport	Grip Force Control	Coordination
Force Rate Spectral Arc Length Transport	Grip Force Control	Coordination
Force Rate Spectral Arc Length Hole Approach	Grip Force Control	Coordination

Supplementary Table S3. Median and interquartile range (IQR) for participant ratings, reported separately for patients (P) and healthcare practitioners (HCP) across all concepts and categories of feedback session 1. Abbreviations: S, screen; V, visual appeal; I, informativeness; U, understandability

**Screen**	**Concept**	**Category**	**Role**	**Median [IQR]**
S1	A1	V	P	4.0 [3.0–5.0]
S1	A1	V	HCP	3.5 [3.0–4.2]
S1	A1	I	P	3.0 [3.0–5.0]
S1	A1	I	HCP	3.0 [3.0–3.0]
S1	A1	U	P	4.0 [3.0–5.0]
S1	A1	U	HCP	5.0 [4.0–5.0]
S1	A2	V	P	5.0 [4.0–5.0]
S1	A2	V	HCP	3.5 [2.8–4.0]
S1	A2	I	P	5.0 [4.0–5.0]
S1	A2	I	HCP	3.0 [3.0–3.2]
S1	A2	U	P	5.0 [4.0–5.0]
S1	A2	U	HCP	3.0 [2.8–3.5]
S1	A3	V	P	5.0 [4.0–5.0]
S1	A3	V	HCP	5.0 [3.0–5.0]
S1	A3	I	P	4.0 [4.0–5.0]
S1	A3	I	HCP	4.5 [3.5–5.0]
S1	A3	U	P	5.0 [4.0–5.0]
S1	A3	U	P	4.5 [3.8–5.0]
S2	B1	V	P	5.0 [4.0–5.0]
S2	B1	V	HCP	5.0 [4.0–5.0]
S2	B1	I	P	5.0 [4.0–5.0]
S2	B1	I	HCP	4.0 [3.8–4.2]
S2	B1	U	P	5.0 [4.0–5.0]
S2	B1	U	HCP	5.0 [4.0–5.0]
S2	B2	V	P	5.0 [5.0–5.0]
S2	B2	V	HCP	4.0 [2.8–4.2]
S2	B2	I	P	5.0 [5.0–5.0]
S2	B2	I	HCP	4.0 [4.0–4.2]
S2	B2	U	P	5.0 [5.0–5.0]
S2	B2	U	HCP	4.0 [4.0–5.0]
S2	B3	V	P	3.0 [2.0–4.0]
S2	B3	V	HCP	3.0 [2.0–3.2]
S2	B3	I	P	3.0 [2.0–4.0]
S2	B3	I	HCP	4.0 [2.8–4.2]
S2	B3	U	P	4.0 [3.0–4.0]
S2	B3	U	HCP	4.0 [3.0–5.0]
S2	B4	V	P	3.0 [2.0–3.0]
S2	B4	V	HCP	3.0 [2.8–3.2]
S2	B4	I	P	2.0 [2.0–3.0]
S2	B4	I	HCP	3.5 [2.8–4.0]
S2	B4	U	P	3.0 [2.0–3.0]
S2	B4	U	HCP	3.0 [2.5–4.5]
S3	C1	V	P	4.0 [3.0–4.0]
S3	C1	V	HCP	4.0 [4.0–5.0]

Screen	Concept	Category	Role	Median [IQR]
S3	C1	I	P	4.0 [3.0–5.0]
S3	C1	I	HCP	4.0 [4.0–4.2]
S3	C1	U	P	5.0 [4.0–5.0]
S3	C1	U	HCP	5.0 [4.0–5.0]
S3	C2	V	P	4.0 [4.0–5.0]
S3	C2	V	HCP	5.0 [4.0–5.0]
S3	C2	I	P	5.0 [5.0–5.0]
S3	C2	I	HCP	5.0 [4.0–5.0]
S3	C2	U	P	5.0 [4.0–5.0]
S3	C2	U	HCP	5.0 [4.0–5.0]

Supplementary Table S4. Median and interquartile range (IQR) for participant ratings, reported separately for patients (P) and healthcare practitioners (HCP) across all concepts of feedback session 2. Abbreviations: S, set; V, visual appeal; I, informativeness; U, understandability

Screen	Concept	Category	Role	Median [IQR]
S1	A1	V	P	4.0 [3.2–4.8]
S1	A1	V	HCP	3.0 [2.0–5.0]
S1	A1	I	P	3.5 [3.0–4.8]
S1	A1	I	HCP	4.0 [3.0–4.0]
S1	A1	U	P	3.5 [3.0–4.0]
S1	A1	U	HCP	4.0 [3.0–4.0]
S1	A2	V	P	4.0 [4.0–4.0]
S1	A2	V	HCP	4.0 [2.0–4.0]
S1	A2	I	P	3.5 [3.0–4.0]
S1	A2	I	HCP	4.0 [3.0–5.0]
S1	A2	U	P	3.0 [2.2–3.0]
S1	A2	U	HCP	4.0 [3.0–4.0]
S1	A3	V	P	4.5 [4.0–5.0]
S1	A3	V	HCP	4.0 [4.0–5.0]
S1	A3	I	P	5.0 [2.8–5.0]
S1	A3	I	HCP	4.0 [2.0–4.0]
S1	A3	U	P	4.0 [3.0–5.0]
S1	A3	U	HCP	4.0 [4.0–5.0]
S2	B1	V	P	4.0 [3.2–4.8]
S2	B1	V	HCP	3.0 [3.0–4.0]
S2	B1	I	P	4.5 [4.0–5.0]
S2	B1	I	HCP	4.0 [3.0–5.0]
S2	B1	U	P	5.0 [3.5–5.0]
S2	B1	U	HCP	3.0 [2.0–5.0]
S2	B2	V	P	4.0 [4.0–4.8]
S2	B2	V	HCP	4.0 [3.0–4.0]
S2	B2	I	P	4.5 [3.2–5.0]
S2	B2	I	HCP	4.0 [3.0–4.0]
S2	B2	U	P	4.0 [3.2–4.8]
S2	B2	U	HCP	4.0 [3.0–5.0]
S2	B3	V	P	4.5 [3.2–5.0]

**Screen**	**Concept**	**Category**	**Role**	**Median [IQR]**
S2	B3	V	HCP	4.0 [3.0–4.0]
S2	B3	I	P	3.5 [3.0–4.8]
S2	B3	I	HCP	4.0 [3.0–5.0]
S2	B3	U	P	3.5 [3.0–4.8]
S2	B3	U	HCP	4.0 [3.0–5.0]
S2	B4	V	P	4.5 [4.0–5.0]
S2	B4	V	HCP	4.0 [3.0–5.0]
S2	B4	I	P	5.0 [3.5–5.0]
S2	B4	I	HCP	4.0 [4.0–5.0]
S2	B4	U	P	5.0 [4.2–5.0]
S2	B4	U	HCP	3.0 [3.0–5.0]
S2	B5	V	P	4.5 [2.5–5.0]
S2	B5	V	HCP	3.0 [2.0–4.0]
S2	B5	I	P	5.0 [4.2–5.0]
S2	B5	I	HCP	3.5 [2.8–4.0]
S2	B5	U	P	5.0 [3.5–5.0]
S2	B5	U	HCP	3.0 [3.0–4.0]
S3	C1	V	P	3.5 [2.2–4.0]
S3	C1	V	HCP	3.0 [3.0–4.0]
S3	C2	V	P	4.0 [4.0–4.8]
S3	C2	V	HCP	4.0 [3.0–4.0]
S3	C3	V	P	3.5 [2.2–4.8]
S3	C3	V	HCP	4.0 [3.0–4.0]
S3	C1	M	P	3.0 [2.0–4.0]
S3	C1	M	HCP	3.0 [3.0–4.0]
S3	C2	M	P	4.0 [4.0–5.0]
S3	C2	M	HCP	4.0 [3.0–5.0]
S3	C3	M	P	4.0 [3.0–5.0]
S3	C3	M	HCP	3.0 [2.0–4.0]

References

[1] R J Butland, J Pang, E R Gross, A A Woodcock, and D M Geddes. Two-, six-, and 12-minute walking tests in respiratory disease. *BMJ*, 284:1607–1608, 5 1982. ISSN 0959-8138. doi: 10.1136/bmj.284.6329.1607.

[2] C Collin and D Wade. Assessing motor impairment after stroke: a pilot reliability study. *Journal of Neurology, Neurosurgery & Psychiatry*, 53:576–579, 7 1990. ISSN 0022-3050. doi: 10.1136/jnnp.53.7.576.

[3] EuroQol Group. Euroqol - a new facility for the measurement of health-related quality of life. *Health Policy*, 16:199–208, 12 1990. ISSN 01688510. doi: 10.1016/0168-8510(90)90421-9.

[4] G H Guyatt, M J Sullivan, P J Thompson, E L Fallen, S O Pugsley, D W Taylor, and L B Berman. The 6-minute walk: a new measure of exercise capacity in patients with chronic heart failure. *Canadian Medical Association journal*, 132:919–23, 4 1985. ISSN 0008-4409.

[5] Ron D. Hays, Jakob B. Bjorner, Dennis A. Revicki, Karen L. Spritzer, and David Cella. Development of physical and mental health summary scores from the patient-reported outcomes measurement information system (promis) global items. *Quality of Life Research*, 18:873–880, 9 2009. ISSN 0962-9343. doi: 10.1007/s11136-009-9496-9.

[6] Maureen K. Holden, Kathleen M. Gill, Marie R. Magliozzi, John Nathan, and Linda Piehl-Baker. Clinical gait assessment in the neurologically impaired. *Physical Therapy*, 64:35–40, 1 1984. ISSN 0031-9023. doi: 10.1093/ptj/64.1.35.

[7] R A Keith, C V Granger, B B Hamilton, and F S Sherwin. The functional independence measure: a new tool for rehabilitation. *Advances in clinical rehabilitation*, 1:6–18, 1987. ISSN 0892-8878.

[8] Marc Kosak and Teresa Smith. Comparison of the 2-, 6-, and 12-minute walk tests in patients with stroke. *The Journal of Rehabilitation Research and Development*, 41:103, 2004. ISSN 0748-7711. doi: 10.1682/JRRD.2003.11.0171.

[9] J. F. Kurtzke. Rating neurologic impairment in multiple sclerosis: An expanded disability status scale (edss). *Neurology*, 33:1444–1444, 11 1983. ISSN 0028-3878. doi: 10.1212/WNL.33.11.1444.

[10] BERNARD S. LINN, MARGARET W. LINN, and LEE GUREL. Cumulative illness rating scale. *Journal of the American Geriatrics Society*, 16:622–626, 5 1968. ISSN 0002-8614. doi: 10.1111/j.1532-5415.1968.tb02103.x.

[11] V Mathiowetz, N Kashman, G Volland, K Weber, M Dowe, and S Rogers. Grip and pinch strength: normative data for adults. *Archives of physical medicine and rehabilitation*, 66:69–74, 2 1985. ISSN 0003-9993.

[12] V. Mathiowetz, G. Volland, N. Kashman, and K. Weber. Adult norms for the box and block test of manual dexterity. *The American journal of occupational therapy : official publication of the American Occupational Therapy Association*, 39:386–391, 1985. ISSN 0272-9490. doi: 10.5014/AJOT.39.6.386. URL `https://pubmed.ncbi.nlm.nih.gov/3160243/`.

[13] Diane Podsiadlo and Sandra Richardson. The timed "up & go": A test of basic functional mobility for frail elderly persons. *Journal of the American Geriatrics Society*, 39:142–148, 2 1991. ISSN 0002-8614. doi: 10.1111/j.1532-5415.1991.tb01616.x.

[14] D T Wade, V A Wood, A Heller, J Maggs, and R Langton Hewer. Walking after stroke. measurement and recovery over the first 3 months. *Scandinavian journal of rehabilitation medicine*, 19:25–30, 1987. ISSN 0036-5505.
