# Supplementary material for: Digital Platform to Provide Health Data Feedback for Neurorehabilitation Patients: User-Centered Development and Proof-of-Concept Usability Study
Source: JMIR Rehabil Assist Technol. 2026 Jun 17;13:e85072. doi: 10.2196/85072 (PMC13274913; doi:10.2196/85072)
Supplement: Multimedia Appendix 2 [file rehab-v13-e85072-s002.pdf]

Zürich, 17. Januar 2024

Fragebogen Patienten Feedback

Teilnehmer/in

ID	
Alter (in Jahren)	
Geschlecht	
Erlernter Beruf	

Bitte füllen Sie diesen Teil aus **<u>bevor</u>** Sie die verschiedenen Konzepte ansehen.

	Ich stimme überhaupt nicht zu		Neutral		Ich stimme voll zu
	1	2	3	4	5
1. Denken Sie, dass Feedback zu durchgeführten Untersuchungen Ihre Motivation zur Therapie erhöhen könnte?	☐	☐	☐	☐	☐

2. Welche Informationen zu durchgeführten Untersuchungen würden Sie gerne erhalten?	
3. Welches Feedback würden Sie gerne von dem Virtual Peg Insertion Test (VPIT) erhalten?	

xx-------------xx

Bitte schauen Sie sich die verschiedenen Konzepte in Ruhe an und beantworten die nachfolgenden Fragen.

Bildschirm 1

	Ich stimme überhaupt nicht zu		Neutral		Ich stimme voll zu
	1	2	3	4	5
1.1. Finden Sie das Feedback motivierend?					
Konzept A	◻	◻	◻	◻	◻
Konzept B	◻	◻	◻	◻	◻
Konzept C	◻	◻	◻	◻	◻
1.2. Finden Sie die Darstellung visuell ansprechend?					
Konzept A	◻	◻	◻	◻	◻
Konzept B	◻	◻	◻	◻	◻
Konzept C	◻	◻	◻	◻	◻
1.3. Finden Sie die Erklärungen informativ?					
Konzept A	◻	◻	◻	◻	◻
Konzept B	◻	◻	◻	◻	◻
Konzept C	◻	◻	◻	◻	◻
1.4. Finden Sie die Erklärungen verständlich dargestellt?					
Konzept A	◻	◻	◻	◻	◻
Konzept B	◻	◻	◻	◻	◻
Konzept C	◻	◻	◻	◻	◻

1.5. Was in dem jeweiligen Konzept ist <u>nicht</u> verständlich dargestellt?	
Konzept A	
Konzept B	
Konzept C	

1.6. Was gefällt Ihnen an dem jeweiligen Konzept?	
Konzept A	
Konzept B	
Konzept C	
1.7. Was gefällt Ihnen <u>nicht</u> an dem jeweiligen Konzept?	
Konzept A	
Konzept B	
Konzept C	
1.8. Was würden Sie an dem jeweiligen Konzept ändern oder hinzufügen wollen?	
Konzept A	
Konzept B	
Konzept C	

1.9. Bitte bewerten Sie die Konzepte indem Sie A, B und C in die Felder eintragen.<br>(1 = bestes Konzept)	1.	2.	3.

Bildschirm 2

	Ich stimme überhaupt nicht zu		Neutral		Ich stimme voll zu
	1	2	3	4	5
2.1. Finden Sie das Feedback motivierend?					
Konzept A	◻	◻	◻	◻	◻
Konzept B	◻	◻	◻	◻	◻
Konzept C	◻	◻	◻	◻	◻
Konzept D	◻	◻	◻	◻	◻
2.2. Finden Sie die Darstellung visuell ansprechend?					
Konzept A	◻	◻	◻	◻	◻
Konzept B	◻	◻	◻	◻	◻
Konzept C	◻	◻	◻	◻	◻
Konzept D	◻	◻	◻	◻	◻
2.3. Finden Sie das Feedback informativ?					
Konzept A	◻	◻	◻	◻	◻
Konzept B	◻	◻	◻	◻	◻
Konzept C	◻	◻	◻	◻	◻
Konzept D	◻	◻	◻	◻	◻
2.4. Finden Sie das Feedback verständlich dargestellt?					
Konzept A	◻	◻	◻	◻	◻
Konzept B	◻	◻	◻	◻	◻
Konzept C	◻	◻	◻	◻	◻
Konzept D	◻	◻	◻	◻	◻

2.5. Was in dem jeweiligen Konzept ist <u>nicht</u> verständlich dargestellt?	
Konzept A	
Konzept B	
Konzept C	

Konzept D	
2.6. Was gefällt Ihnen an dem jeweiligen Konzept?	
Konzept A	
Konzept B	
Konzept C	
Konzept D	
2.7. Was gefällt Ihnen <u>nicht</u> an dem jeweiligen Konzept?	
Konzept A	
Konzept B	
Konzept C	
Konzept D	
2.8.. Was würden Sie an dem jeweiligen Konzept ändern oder hinzufügen wollen?	
Konzept A	
Konzept B	
Konzept C	
Konzept D	

2.9. Bitte bewerten Sie die Konzepte indem Sie A, B C und D in die Felder eintragen. (1 = bestes Konzept)	1.	2.	3.	4.

Bildschirm 3

	Ich stimme überhaupt nicht zu		Neutral		Ich stimme voll zu
	1	2	3	4	5
3.1. Finden Sie die Darstellung visuell ansprechend?					
Konzept A	☐	☐	☐	☐	☐
Konzept B	☐	☐	☐	☐	☐
3.2. Finden Sie das Feedback informativ?					
Konzept A	☐	☐	☐	☐	☐
Konzept B	☐	☐	☐	☐	☐
3.3. Finden Sie das Feedback verständlich dargestellt?					
Konzept A	☐	☐	☐	☐	☐
Konzept B	☐	☐	☐	☐	☐

3.4. Was in dem jeweiligen Konzept ist <u>nicht</u> verständlich dargestellt?	
Konzept A	
Konzept B	
3.5. Was gefällt Ihnen an dem jeweiligen Konzept?	
Konzept A	
Konzept B	
3.6. Was gefällt Ihnen <u>nicht</u> an dem jeweiligen Konzept?	
Konzept A	
Konzept B	

3.7. Was würden Sie an dem jeweiligen Konzept ändern oder hinzufügen wollen?	
Konzept A	
Konzept B	

Bitte bewerten Sie die Konzepte indem Sie A, und B in die Felder eintragen. (1 = bestes Konzept)	1.	2.

Allgemein

	Ich stimme überhaupt nicht zu		Neutral		Ich stimme voll zu
	1	2	3	4	5
1. Denken Sie, dass Feedback zu durchgeführten Untersuchungen Ihre Motivation zur Therapie erhöhen könnte?	☐	☐	☐	☐	☐

Hier ist Platz für Anmerkungen, Ideen oder Kommentare zu Feedback und dem VPIT.
