# Supplementary material for: Digital Platform to Provide Health Data Feedback for Neurorehabilitation Patients: User-Centered Development and Proof-of-Concept Usability Study
Source: JMIR Rehabil Assist Technol. 2026 Jun 17;13:e85072. doi: 10.2196/85072 (PMC13274913; doi:10.2196/85072)
Supplement: Multimedia Appendix 3 [file rehab-v13-e85072-s003.pdf]

Zürich, 17. Januar 2024

Fragebogen Therapeuten Feedback

Teilnehmer/in

ID	
Alter (in Jahren)	
Geschlecht	
Erlernter Beruf	

Bitte füllen Sie diesen Teil aus **bevor** Sie die verschiedenen Konzepte ansehen.

	Ich stimme überhaupt nicht zu		Neutral		Ich stimme voll zu
	1	2	3	4	5
1. Könnte Ihnen Feedback zu durchgeführten Untersuchungen bei der Entscheidungsfindung behilflich sein?	☐	☐	☐	☐	☐
2. Könnte Ihnen Feedback zu durchgeführten Untersuchungen einen Mehrwert bringen?	☐	☐	☐	☐	☐
3. Denken Sie, dass Feedback zu durchgeführten Untersuchungen motivierend für Patienten sein könnte?	☐	☐	☐	☐	☐

4. Welche Informationen zu durchgeführten Untersuchungen würden Sie gerne erhalten?	
5. Welches Feedback würden Sie gerne von dem Virtual Peg Insertion Test (VPIT) erhalten?	

Bitte schauen Sie sich die verschiedenen Konzepte in Ruhe an und beantworten die nachfolgenden Fragen.

Bildschirm 1

	Ich stimme überhaupt nicht zu		Neutral		Ich stimme voll zu
	1	2	3	4	5
1.1 Könnte Ihnen das dargestellte Feedback bei der Entscheidungsfindung behilflich sein?					
Konzept A	☐	☐	☐	☐	☐
Konzept B	☐	☐	☐	☐	☐
Konzept C	☐	☐	☐	☐	☐
1.2. Denken Sie, dass das Feedback für Patienten motivierend ist?					
Konzept A	☐	☐	☐	☐	☐
Konzept B	☐	☐	☐	☐	☐
Konzept C	☐	☐	☐	☐	☐
1.3. Finden Sie die Darstellung visuell ansprechend?					
Konzept A	☐	☐	☐	☐	☐
Konzept B	☐	☐	☐	☐	☐
Konzept C	☐	☐	☐	☐	☐
1.4. Finden Sie das Feedback informativ?					
Konzept A	☐	☐	☐	☐	☐
Konzept B	☐	☐	☐	☐	☐
Konzept C	☐	☐	☐	☐	☐
1.5. Finden Sie das Feedback verständlich dargestellt?					
Konzept A	☐	☐	☐	☐	☐
Konzept B	☐	☐	☐	☐	☐
Konzept C	☐	☐	☐	☐	☐

1.6. Was in dem jeweiligen Konzept ist <u>nicht</u> verständlich dargestellt?	
Konzept A	

Konzept B	
Konzept C	
1.7. Was gefällt Ihnen an dem jeweiligen Konzept?	
Konzept A	
Konzept B	
Konzept C	
1.8. Was gefällt Ihnen <u>nicht</u> an dem jeweiligen Konzept?	
Konzept A	
Konzept B	
Konzept C	
1.9. Was würden Sie an dem jeweiligen Konzept ändern oder hinzufügen wollen?	
Konzept A	
Konzept B	
Konzept C	

1.10. Bitte bewerten Sie die Konzepte indem Sie A, B und C in die Felder eintragen. (1 = bestes Konzept)	1.	2.	3.

Bildschirm 2

	Ich stimme überhaupt nicht zu		Neutral		Ich stimme voll zu
	1	2	3	4	5
2.1 Könnte Ihnen das dargestellte Feedback bei der Entscheidungsfindung behilflich sein?					
Konzept A	◻	◻	◻	◻	◻
Konzept B	◻	◻	◻	◻	◻
Konzept C	◻	◻	◻	◻	◻
Konzept D	◻	◻	◻	◻	◻
2.2. Denken Sie, dass das Feedback für Patienten motivierend ist?					
Konzept A	◻	◻	◻	◻	◻
Konzept B	◻	◻	◻	◻	◻
Konzept C	◻	◻	◻	◻	◻
Konzept D	◻	◻	◻	◻	◻
2.3. Finden Sie die Darstellung visuell ansprechend?					
Konzept A	◻	◻	◻	◻	◻
Konzept B	◻	◻	◻	◻	◻
Konzept C	◻	◻	◻	◻	◻
Konzept D	◻	◻	◻	◻	◻
2.4. Finden Sie das Feedback informativ?					
Konzept A	◻	◻	◻	◻	◻
Konzept B	◻	◻	◻	◻	◻
Konzept C	◻	◻	◻	◻	◻
Konzept D	◻	◻	◻	◻	◻
2.5. Finden Sie das Feedback verständlich dargestellt?					
Konzept A	◻	◻	◻	◻	◻
Konzept B	◻	◻	◻	◻	◻
Konzept C	◻	◻	◻	◻	◻
Konzept D	◻	◻	◻	◻	◻

2.6. Was in dem jeweiligen Konzept ist <u>nicht</u> verständlich dargestellt?	
Konzept A	
Konzept B	
Konzept C	
Konzept D	
2.7. Was gefällt Ihnen an dem jeweiligen Konzept?	
Konzept A	
Konzept B	
Konzept C	
Konzept D	
2.8. Was gefällt Ihnen <u>nicht</u> an dem jeweiligen Konzept?	
Konzept A	
Konzept B	
Konzept C	
Konzept D	
2.9.. Was würden Sie an dem jeweiligen Konzept ändern oder hinzufügen wollen?	
Konzept A	

Konzept B	
Konzept C	
Konzept D	

2.10. Bitte bewerten Sie die Konzepte indem Sie A, B C und D in die Felder eintragen. (1 = bestes Konzept)	1.	2.	3.	4.

Bildschirm 3

	Ich stimme überhaupt nicht zu		Neutral		Ich stimme voll zu
	1	2	3	4	5
3.1. Finden Sie die Darstellung visuell ansprechend?					
Konzept A	☐	☐	☐	☐	☐
Konzept B	☐	☐	☐	☐	☐
3.2. Finden Sie das Feedback informativ?					
Konzept A	☐	☐	☐	☐	☐
Konzept B	☐	☐	☐	☐	☐
3.3. Finden Sie das Feedback verständlich dargestellt?					
Konzept A	☐	☐	☐	☐	☐
Konzept B	☐	☐	☐	☐	☐

3.4. Was in dem jeweiligen Konzept ist <u>nicht</u> verständlich dargestellt?	
Konzept A	
Konzept B	
3.5. Was gefällt Ihnen an dem jeweiligen Konzept?	
Konzept A	
Konzept B	
3.6. Was gefällt Ihnen <u>nicht</u> an dem jeweiligen Konzept?	
Konzept A	
Konzept B	

3.7. Was würden Sie an dem jeweiligen Konzept ändern oder hinzufügen wollen?	
Konzept A	
Konzept B	

3.8. Bitte bewerten Sie die Konzepte indem Sie A, und B in die Felder eintragen.<br>(1 = bestes Konzept)	1.	2.

Allgemein

	Ich stimme überhaupt nicht zu		Neutral		Ich stimme voll zu
	1	2	3	4	5
1. Könnte Ihnen Feedback zu durchgeführten Untersuchungen bei der Entscheidungsfindung behilflich sein?	☐	☐	☐	☐	☐
2. Denken Sie, dass das VPIT bei der Bewertung der Funktion der oberen Extremitäten hilfreich sein könnte?	☐	☐	☐	☐	☐

Hier ist Platz für Anmerkungen, Ideen oder Kommentare zu Feedback und dem VPIT.
