# Supplementary material for: Digital Platform to Provide Health Data Feedback for Neurorehabilitation Patients: User-Centered Development and Proof-of-Concept Usability Study
Source: JMIR Rehabil Assist Technol. 2026 Jun 17;13:e85072. doi: 10.2196/85072 (PMC13274913; doi:10.2196/85072)
Supplement: Multimedia Appendix 4 [file rehab-v13-e85072-s004.pdf]

Zürich, 20. März 2024

Fragebogen Patienten Feedback 2

Teilnehmer/in

ID	
Alter (in Jahren)	
Geschlecht	
Erlernter Beruf	

Bitte schauen Sie sich die verschiedenen Konzepte in Ruhe an und beantworten die nachfolgenden Fragen.

1 Darstellung Erklärungen

	Ich stimme überhaupt nicht zu		Neutral		Ich stimme voll zu
	1	2	3	4	5
1.1 Finden Sie die Erklärung verständlich dargestellt?					
Konzept A	☐	☐	☐	☐	☐
Konzept B	☐	☐	☐	☐	☐
Konzept C	☐	☐	☐	☐	☐
1.2. Finden Sie die Erklärungen informativ?					
Konzept A	☐	☐	☐	☐	☐
Konzept B	☐	☐	☐	☐	☐
Konzept C	☐	☐	☐	☐	☐
1.3. Finden Sie die Darstellung visuell ansprechend?					
Konzept A	☐	☐	☐	☐	☐
Konzept B	☐	☐	☐	☐	☐
Konzept C	☐	☐	☐	☐	☐
1.4. Finden Sie die Erklärungen motivierend?					
Konzept A	☐	☐	☐	☐	☐
Konzept B	☐	☐	☐	☐	☐
Konzept C	☐	☐	☐	☐	☐

1.5. Was in dem jeweiligen Konzept ist nicht verständlich dargestellt?	
Konzept A	
Konzept B	
Konzept C	

1.6. Was gefällt Ihnen an dem jeweiligen Konzept?	
Konzept A	
Konzept B	
Konzept C	
1.7. Was gefällt Ihnen nicht an dem jeweiligen Konzept?	
Konzept A	
Konzept B	
Konzept C	
1.8. Was würden Sie an dem jeweiligen Konzept ändern oder hinzufügen wollen?	
Konzept A	
Konzept B	
Konzept C	

1.9. Bitte bewerten Sie die Konzepte indem Sie A, B und C in die Felder eintragen.<br>(1 = bestes Konzept)	1.	2.	3.

Bitte schauen Sie sich die verschiedenen Konzepte in Ruhe an und beantworten die nachfolgenden Fragen.

2 Erklärungen VPIT Messwerte

	Ich stimme überhaupt nicht zu		Neutral		Ich stimme voll zu
	1	2	3	4	5
2.1. Finden Sie die Erklärung verständlich dargestellt?					
Konzept A	☐	☐	☐	☐	☐
Konzept B	☐	☐	☐	☐	☐
Konzept C	☐	☐	☐	☐	☐
Konzept D	☐	☐	☐	☐	☐
Konzept E	☐	☐	☐	☐	☐
2.2. Finden Sie die Erklärungen informativ?					
Konzept A	☐	☐	☐	☐	☐
Konzept B	☐	☐	☐	☐	☐
Konzept C	☐	☐	☐	☐	☐
Konzept D	☐	☐	☐	☐	☐
Konzept E	☐	☐	☐	☐	☐
2.3. Finden Sie die Darstellung visuell ansprechend?					
Konzept A	☐	☐	☐	☐	☐
Konzept B	☐	☐	☐	☐	☐
Konzept C	☐	☐	☐	☐	☐
Konzept D	☐	☐	☐	☐	☐
Konzept E	☐	☐	☐	☐	☐
2.4. Finden Sie die Erklärungen motivierend?					
Konzept A	☐	☐	☐	☐	☐
Konzept B	☐	☐	☐	☐	☐
Konzept C	☐	☐	☐	☐	☐
Konzept D	☐	☐	☐	☐	☐
Konzept E	☐	☐	☐	☐	☐

2.5. Was in dem jeweiligen Konzept ist <u>nicht</u> verständlich dargestellt?	
Konzept A	
Konzept B	
Konzept C	
Konzept D	
Konzept E	
2.6. Was gefällt Ihnen an dem jeweiligen Konzept?	
Konzept A	
Konzept B	
Konzept C	
Konzept D	
Konzept E	
2.7. Was gefällt Ihnen <u>nicht</u> an dem jeweiligen Konzept?	
Konzept A	
Konzept B	
Konzept C	
Konzept D	
Konzept E	

2.8. Was würden Sie an dem jeweiligen Konzept ändern oder hinzufügen wollen?	
Konzept A	
Konzept B	
Konzept C	
Konzept D	
Konzept E	

Bitte schauen Sie sich die verschiedenen Konzepte in Ruhe an und beantworten die nachfolgenden Fragen.

3 Darstellung Messwerte

	Ich stimme überhaupt nicht zu		Neutral		Ich stimme voll zu
	1	2	3	4	5
3.1. Finden Sie das Feedback motivierend?					
Konzept A	☐	☐	☐	☐	☐
Konzept B	☐	☐	☐	☐	☐
Konzept C	☐	☐	☐	☐	☐
3.2. Finden Sie das Feedback visuell ansprechend?					
Konzept A	☐	☐	☐	☐	☐
Konzept B	☐	☐	☐	☐	☐
Konzept C	☐	☐	☐	☐	☐
3.3. Finden Sie den Text verständlich formuliert?	☐	☐	☐	☐	☐
3.4. Finden Sie den Text motivierend?	☐	☐	☐	☐	☐

3.5. Was gefällt Ihnen an dem jeweiligen Konzept?	
Konzept A	
Konzept B	
Konzept C	
3.6. Was gefällt Ihnen <u>nicht</u> an dem jeweiligen Konzept?	
Konzept A	
Konzept B	
Konzept C	

3.8. Was würden Sie an dem jeweiligen Konzept ändern oder hinzufügen wollen?	
Konzept A	
Konzept B	
Konzept C	
3.8. Würden Sie an dem Text etwas ändern?<br>Wenn ja, was würden Sie ändern?	

3.9. Bitte bewerten Sie die Konzepte indem Sie A, B und C in die Felder eintragen.<br>(1 = bestes Konzept)	1.	2.	3.

Hier ist Platz für Anmerkungen, Ideen oder Kommentare zu Feedback und dem VPIT.
