# Supplementary material for: Digital Platform to Provide Health Data Feedback for Neurorehabilitation Patients: User-Centered Development and Proof-of-Concept Usability Study
Source: JMIR Rehabil Assist Technol. 2026 Jun 17;13:e85072. doi: 10.2196/85072 (PMC13274913; doi:10.2196/85072)
Supplement: Multimedia Appendix 6 [file rehab-v13-e85072-s006.pdf]

Zürich, 20. Mai 2024

Fragebogen Patienten Feedback 3

Teilnehmer*in

ID	
Alter (in Jahren)	
Geschlecht	
Erlernter Beruf	

1 Fragen Systembedienung

	Ich stimme überhaupt nicht zu		Neutral		Ich stimme voll zu
	1	2	3	4	5
1.1 Ich denke, dass ich dieses Produkt häufiger verwenden möchte.	◻	◻	◻	◻	◻
1.2. Ich fand das Produkt unnötig komplex.	◻	◻	◻	◻	◻
1.3. Ich dachte, das Produkt war einfach zu bedienen.	◻	◻	◻	◻	◻
1.4. Ich denke, dass ich die Unterstützung einer technischen Person brauche, um dieses Produkt nutzen zu können.	◻	◻	◻	◻	◻
1.5 Ich fand, die verschiedenen Funktionen in diesem Produkt waren gut integriert.	◻	◻	◻	◻	◻
1.6 Ich dachte, dass dieses Produkt nicht konsistent genug war.	◻	◻	◻	◻	◻
1.7 Ich würde mir vorstellen, dass die meisten Leute sehr schnell lernen würden, dieses Produkt zu benutzen.	◻	◻	◻	◻	◻
1.8 Ich fand dieses Produkt sehr umständlich zu benutzen.	◻	◻	◻	◻	◻
1.9 Ich habe mich sehr selbstsicher gefühlt, dieses Produkt zu verwenden.	◻	◻	◻	◻	◻
1.10 Ich musste eine Menge Dinge lernen, bevor ich mit diesem Produkt loslegen konnte.	◻	◻	◻	◻	◻

2 Allgemeine Fragen

	Ich stimme überhaupt nicht zu		Neutral		Ich stimme voll zu
	1	2	3	4	5
2.1 Denken Sie, dass Feedback zu durchgeführten Untersuchungen Ihre Motivation zur Therapie erhöhen könnte?	☐	☐	☐	☐	☐
2.2 Die App enthält alle Informationen zum VPIT, die ich gerne erhalten würde.	☐	☐	☐	☐	☐
2.3 Die App enthält alle Informationen zu durchgeführten Untersuchungen, die ich gerne erhalten würde.	☐	☐	☐	☐	☐
2.4 Ich würde gerne wissen, wie ich in den Tests im Vergleich zu Menschen ohne Beeinträchtigung abschneide.	☐	☐	☐	☐	☐
2.5 Ich würde gerne wissen, wie ich in den Tests im Vergleich zu anderen Patient*innen in der Klinik abschneide.	☐	☐	☐	☐	☐
2.6 Ich würde das Erreichen persönlicher Meilensteine gerne (mehr) feiern.	☐	☐	☐	☐	☐

2.7 Würden Sie an der App etwas ändern wollen? Was wäre das?	
2.8 Würden Sie etwas zu der App hinzufügen, oder aus der App entfernen wollen? Was wäre das?	

Hier ist Platz für Anmerkungen, Ideen oder Kommentare zum Feedback und dem VPIT.
